# Differentiation strategies for planktonic bacteria and eukaryotes in response to aggravated algal blooms in urban lakes

**DOI:** 10.1002/imt2.84

**Published:** 2023-01-31

**Authors:** Wenjie Wan, Hans‐Peter Grossart, Donglan He, Wenzhi Liu, Shuai Wang, Yuyi Yang

**Affiliations:** ^1^ Key Laboratory of Aquatic Botany and Watershed Ecology Wuhan Botanical Garden Chinese Academy of Sciences Wuhan People's Republic of China; ^2^ College of Life Science South‐Central Minzu University Wuhan People's Republic of China; ^3^ Departent of Plankton and Microbial Ecology Leibniz‐Institute for Freshwater Ecology and Inland Fisheries (IGB) Neuglobsow Germany; ^4^ Institute of Biochemistry and Biology University of Potsdam Potsdam Germany; ^5^ Danjiangkou Wetland Ecosystem Field Scientific Observation and Research Station Chinese Academy of Sciences & Hubei Province Wuhan People's Republic of China

## Abstract

Aggravated algal blooms potentially decreased environmental heterogeneity. Different strategies of planktonic bacteria and eukaryotes in response to aggravated algal blooms. Environmental constraints of plankton showed different patterns over time.

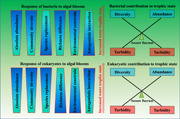

## INTRODUCTION

Protecting ecological health of waterbodies for drinking, agricultural, and industrial utilization caters to one of the current global sustainable development goals (SDG, https://sdgs.un.org/goals) [1]. Many measures (e.g., dredging) have been taken to promise good waterbody quality by depleting nutrients (e.g., phosphorus and nitrogen) availability [2–4]. However, algal blooms, especially harmful cyanobacterial blooms caused mainly by eutrophication, occur seasonally and periodically in inland and coastal aquatic ecosystems despite strict control of external nutrient loading [5, 6]. Previous studies have reported that algal blooms, for example, *Microcystis* blooms, threaten planktonic diversity by releasing secondary metabolites (e.g., algal toxins) and consuming oxygen [5, 7]. Therefore, it is of great importance to disentangle how plankton respond to algal blooms and factors controlling water trophic state.

Typically, the key parameters (e.g., chlorophyll‐*α* [Chl‐*α*] plus total phosphorus, total nitrogen, and/or chemical oxygen demand) are used to reflect and predict the trophic level of waterbody [8]. Nutritional factors (e.g., nitrogen and phosphorus) and nonnutritional factors (e.g., temperature and turbidity) influence Chl‐*α* content [9, 10]. For example, reducing phosphorus level can mitigate cyanobacterial density in a hyper‐eutrophic lake [9]. Microorganisms mediate nitrogen and phosphorus transformation in waters and sediments, in particular in the absence of oxygen [11, 12]. Bacteria (e.g., cyanobacteria and actinobacteria) and eukaryotes (e.g., phytoplankton, zooplankton, and protozoa) are important components of aquatic food webs [13], and planktonic interaction affects biomass of Chl‐*α*‐containing plankton and planktonic diversity [14, 15]. For example, actinomycete display algicidal effect on *Alexandrium tamarense* [14], and flagellates can degrade toxic *Microcystis* sp. by producing functional compounds (e.g., peroxiredoxin and phosphatase) [15]. Therefore, deciphering water trophic state is essential to investigate the content of Chl‐*α* relying on the biomass of Chl‐*α*‐containing organisms (e.g., prokaryotic cyanobacteria and eukaryotic algae) [8]. Planktonic diversity drives multinutrient (e.g., nitrogen and phosphorus) cycles [16]. However, whether planktonic diversity could potentially affect the water trophic state is poorly understood during aggravated algal blooms. Most studies prefer to investigate community composition and diversity of plankton in different aquatic environments [17–19]. However, it remains largely unknown about mechanisms underlying planktonic diversity maintenance in response to aggravated algal blooms.

Many attempts have been made to simultaneously estimate the diversity maintenance of planktonic prokaryotes and eukaryotes by assessing species presence–absence and abundance, ecological assembly processes, and species coexistence patterns [20–23]. In a community, species presence–absence and abundance can be interpreted by species replacement and abundance difference [20]. Ecological assembly processes include determinism (e.g., species sorting) and stochasticity, with the former imposed by abiotic and biotic factors and the later induced by random events (e.g., birth, death, and/or drift) [24, 25]. Both environmental factors and environmental heterogeneity adjust the balance between stochasticity and determinism [25, 26]. For instance, salinity is the major determinant in shaping community assemblies of bacterioplankton in the Yellow River Estuary [26] and microeukaryotes in urban reservoirs [21]. Environmental heterogeneity determines bacterioplankton community assembly processes (i.e., homogeneous selection and heterogeneous selection) and thus governs community turnover and coexistence patterns in the Paraná River [25]. Coexistence patterns, reflected by co‐occurrence network, can infer species interactions [27, 28]. According to co‐occurrence networks, species can be identified as mutualistic or antagonistic based on positive or negative interactions, as well as hub species balancing community stability [25]. Ecological assembly processes and coexistence patterns of planktonic prokaryotes and eukaryotes are reported for many aquatic environments (e.g., rivers, lakes, and reservoirs) [17, 21, 25, 26, 29], but have been insufficiently studied in urban lakes suffering from massive algal blooms.

In this study, we chose 12 representative urban lakes located in Wuhan City (Supporting Information Table S1 and Figure S1) and we collected water samples in April, May, and June to follow algal bloom development. According to a criterion of defining algal blooms with Chl‐*α* threshold of 40 μg/L [30], algal blooms of these lakes were aggravated (i.e., June > May > April [83.29 ± 67.11 μg/L]; Supporting Information: Figure S2). Here, we aim to (i) explore distribution patterns, species replacement and abundance differences, ecological assembly processes, and coexistence patterns of bacteria and eukaryotes in response to aggravated algal blooms, and (ii) elucidate abiotic and biotic factors affecting water trophic state. Considering that the water physicochemical properties change periodically [17, 22, 31], we hypothesized that distribution patterns, ecological assembly processes, and coexistence patterns of both bacteria and eukaryotes differ among sampling months. Because bacteria and eukaryotes are different organisms with different living styles [13, 16], we hypothesized that bacteria and eukaryotes would display the opposite environmental constraint to aggravated algal blooms. We measured water physicochemical properties and conducted Illumina MiSeq sequencing of bacterial and eukaryotic communities to address our research objectives and verify our research hypothesis. We found different responses from bacterial and eukaryotic communities to aggravated algal blooms.

## RESULTS

### Changes in the water trophic state and environmental heterogeneity over time

Water physicochemical properties varied with sampling month (Supporting Information: Figure S2), showing significant differences in nitrate nitrogen, chemical oxygen demand, calcium, magnesium, iron, electrical conductivity, pH, temperature, and dissolved oxygen (*p* < 0.05). No significant differences were found in turbidity, total phosphorus, soluble reactive phosphorus, total nitrogen, and ammonia nitrogen among the 3 months (*p* > 0.05). The Chl‐*α* content (2.21–564.27 μg/L) notably increased from April to June, and more than 60% lakes displayed algal blooms in three sampling months (i.e., April, 66.7%; May, 70.4%; and June, 77.8%). There were significant increases in trophic lake index (TLI) of these lakes from April (37.11–81.55; mesotrophic‐hypereutrophic) to June (40.35–100.16; mesotrophic‐hypereutrophic) (*p* < 0.05; Supporting Information: Figure S2). Only turbidity was significantly correlated with Chl‐*α* and TLI in all 3 months (*p* < 0.05 or *p* < 0.01 or *p* < 0.001; Supporting Information: Table S2). Environmental heterogeneity was significantly higher in April than in May and June (*p* < 0.05; April > May > June) (Supporting Information: Figure S3). These results indicate that there were aggravated algal blooms and decreased environmental heterogeneity over time.

### General distribution patterns of planktonic bacteria and eukaryotes

Absolute abundances of bacteria and eukaryotes significantly increased from April to June (Supporting Information: Figure S4), and were differently correlated with physicochemical factors (Supporting Information: Table S3). For instance, eukaryotic abundances were notably correlated with turbidity in April (*r* = 0.518, *p* < 0.01), May (*r* = 0.506, *p* < 0.01), and June (*r* = 0.567, *p* < 0.01). Both bacterial and eukaryotic community compositions exhibited distinct differences between the 3 months (Supporting Information: Figure S5). A total of 10,511 bacterial amplicon sequence variants (ASVs) and 4487 eukaryotic ASVs were found in 3 months, and they shared 2080 and 783 ASVs, respectively. Bacterial communities were dominated by *Proteobacteria* (37.22%–59.82%), *Actinobacteria* (18.78%–33.85%), *Bacteroidetes* (5.65%–12.86%), *Firmicutes* (1.51%–5.45%), *Deinococcus*‐*Thermus* (0.32%–3.86%), *Cyanobacteria* (3.08%–14.70%), and *Verrucomicrobia* (0.56%–1.25%) (Supporting Information: Figure S5). The relative abundance of *Cyanobacteria* increased from April to June, and *Cyanobacteria* was significantly positively correlated with Chl‐*α* in 3 sampling months (*p* < 0.05 or *p* < 0.001; Supporting Information: Table S4). In contrast, eukaryotic communities were dominated by *Chlorophyta* (7.69%–20.42%), *Rotifera* (11.43%–13.30%), *Arthropoda* (8.59%–14.02%), *Chytridiomycota* (0.44%–5.16%), *Dinophyceae* (2.50%–6.02%), *Chrysophyceae* (0.71%–5.63%), *Bacillariophyta* (1.48%–7.64%), and *Streptophyta* (0.32%–1.21%). Relative abundances of *Dinophyceae* and *Bacillariophyta* increased from April to June. However, *Chlorophyta* rather than other plankton was significantly correlated with TLI in 3 sampling months (*p* < 0.05 or *p* < 0.001; Supporting Information: Table S4). Nonmetric multidimensional scaling (NMDS) plots showed significant differences in bacterial (pairwise analyses of similarity [ANOSIM], *R* = 0.563, *p* < 0.001) and eukaryotic (ANOSIM, *R* = 0.160, *p* < 0.001) community composition over time. Significant distance‐decay relationships (DDRs) were found for bacteria and eukaryotes between the 3 months (*p* < 0.05 or *p* < 0.01 or *p* < 0.001), but most fitness values were relatively low at taxonomic and phylogenetic levels (*R* ^2^ < 0.1) (Supporting Information: Figure S6), indicating a weak decay of community similarity with geographical distance. The β‐diversities of bacteria and eukaryotes revealed significant differences between the 3 months at taxonomic and phylogenetic levels (Supporting Information: Figure S6). Except in June, water physicochemical factors explained more on community compositional variation for eukaryotes than bacteria based on redundancy analysis (RDA) results (Supporting Information: Figure S7). Based on permutational multivariate analysis of variance (PERMANOVA) results, turbidity showed significant effects on the community composition of bacteria and eukaryotes in 3 months (Supporting Information: Figure S8). These results indicate that there are distinct shifts in distribution patterns of bacteria and eukaryotes over time, and turbidity showed important roles in shaping community composition of bacteria and eukaryotes.

**Figure 1:**
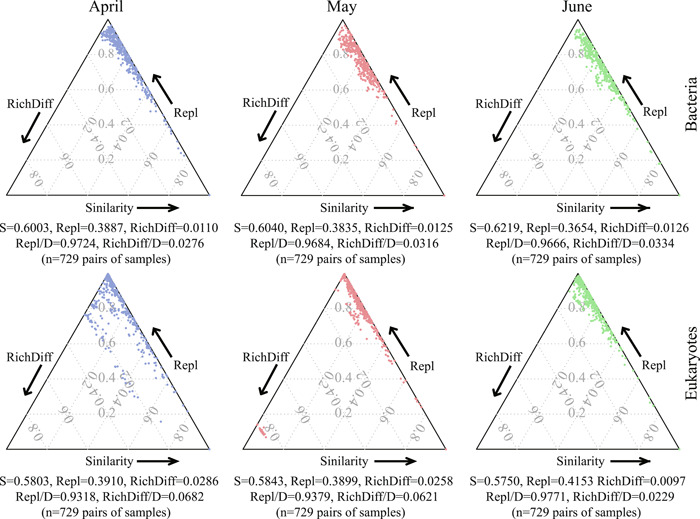
Triangular plots showing community ecological processes (i.e., species replacement and richness difference) of bacteria and eukaryotes in 3 months. Each point (blue, red, and green dots) represents a pair of samples. Its position is determined by a triplet of values from the similarity (S) = 1 – D (dissimilarity), Repl (species replacement), RichDiff (richness difference) matrices; each triplet sums to 1.

By disassembling planktonic taxonomic β‐diversity (Figure 1), we found species replacement showed comparable effects on compositional dissimilarity than richness difference. The ratio of species replacement to compositional dissimilarity (Repl/D) of bacterial community (mean values of Repl/D: April, 0.9724; May, 0.9684; June, 0.9666) significantly decreased over time, but opposite for eukaryotic community (mean values of Repl/D: April, 0.9318; May, 0.9379; June, 0.9771) (*p* < 0.05; Supporting Information: Figure S9). These results indicate the opposite change in trend of species replacement of bacteria and eukaryotes.

Unexpectedly, taxonomic α‐diversity represented by Shannon–Wiener index of bacterial and eukaryotic communities was notably lower in April than in May and June (Supporting Information: Figure S4). This result suggests a distinct increase in planktonic diversity along aggravated algal blooms.

### Ecological community assemblies and co‐occurrence patterns

Mantel correlograms consistently displayed noticeable positive correlations across short phylogenetic distances for bacteria and eukaryotes along environmental gradients from April to June (Supporting Information: Figure S10). Except for the eukaryotes in May, there were also significant negative correlations across short and intermediate phylogenetic distances of bacteria and eukaryotes. The phylogenetic distance covers a significant phylogenetic signal varied from 10% to 40% of the maximum phylogenetic distance within each phylogenetic tree. These results reflect significant phylogenetic signals of bacteria and eukaryotes at short phylogenetic distances along environmental gradients during aggravated algal blooms.

**Figure 2:**
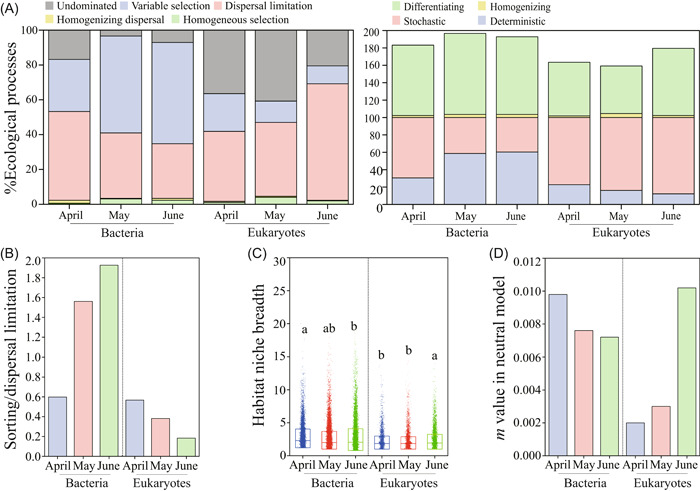
Ecological assembly processes of bacterial and eukaryotic communities in the 3 sampling months. (A) The null model analysis reveals relative contributions of ecological processes to community assemblies of bacteria and eukaryotes in April, May, and June. Environmental constraints of bacteria and eukaryotes based on the species sorting to dispersal limitation ratio (B), habitat niche breadth (C), and migration rate (*m* value) in the neutral model (D). Different letters above columns represent significance (*p* < 0.05).

Subsequently, we evaluated community assemblies of bacteria and eukaryotes over time (Figure 2). Dispersal limitation (31.34%–66.95%), variable selection (10.26%–58.12%), and “undominated” processes (3.42%–40.74%) showed large effects on community assemblies of bacteria and eukaryotes in the 3 months, whereas homogeneous selection (0.57%–3.99%) and homogenizing dispersal (0.28%–1.71%) displayed limited influences in 3 months (Figure 2A). Deterministic (58.67% for May; 60.40% for June) and stochastic (41.31% for May; 39.60% for June) processes balanced bacterial community assembly. Stochastic processes showed major influences on the community assemblies of bacteria in April (69.52%) and eukaryotes in all months (77.21%–87.75%). Generally, differentiating (54.70%–93.17%) rather than homogenizing (1.71%–4.56%) processes dominated the community assemblies of both bacteria and eukaryotes (Figure 2A). These results indicate that stochastic and deterministic processes affected bacterial community assemblies, whereas solely stochastic processes determined eukaryotic community assemblies during aggravated algal blooms.

The ratio of bacterial sorting to dispersal limitation increased over time, but showed an opposite for eukaryotes (Figure 2B). Bacterial habitat niche breadth decreased with time, but the opposite for eukaryotes (Figure 2C). Bacterial migration rates (*m* value) derived from Sloan neutral model decreased with time, whereas an opposite pattern was found for eukaryotes (Figure 2D). These results indicate that bacteria are more environmentally constrained with time, that is, during aggravated algal blooms, and thus behaved the opposite to eukaryotes.

We constructed co‐occurrence networks to decipher the coexistence patterns of bacteria and eukaryotes (Supporting Information: Figure S11 and Table S5). More nodes were found in bacterial and eukaryotic communities in June than in April and May. Ratios of positive edges to negative edges for bacteria increased with time, which was the opposite for eukaryotes (Table S5). This suggests decreasing and increasing potential antagonistic interactions within bacteria and within eukaryotes, respectively. When bacterial and eukaryotic communities were treated as a whole, the number of nodes revealed a similarity (June > May > April) for nodes (Supporting Information: Figure S12 and Table S5). The total planktonic community had a higher ratio of positive to negative edges in June than in April and May (Supporting Information: Table S5), suggesting a decreasing degree of potential antagonistic interactions. The top five core nodes (those with the highest betweenness centralities) in the planktonic conetworks in 3 months were affiliated with *Acinetobacter*, *Candidatus*_*Aquirestis*, *Cryptomonas*, *Deinococcus*, *Limnobacter*, *Limnohabitans*, *Mycobacterium*, and *Rhodoferax* ASVs (Supporting Information: Figure S12). Except for ASV_42847 identified as *Acinetobacter* in April, the relative abundances of these core species were <1% and some even <0.1% (rare taxa) and were related differently to the physicochemical variables (Supporting Information: Figure S12). For instance, the relative abundance of ASV_227008 identified as *Acinetobacter* in April was noticeably correlated with turbidity (*p* < 0.01), dissolved oxygen (*p* < 0.05), and Chl‐*α* (*p* < 0.01). These results support comparable differences in co‐occurrence patterns of bacteria and eukaryotes between the 3 months.

### Effects of biotic and abiotic factors on TLI

**Figure 3:**
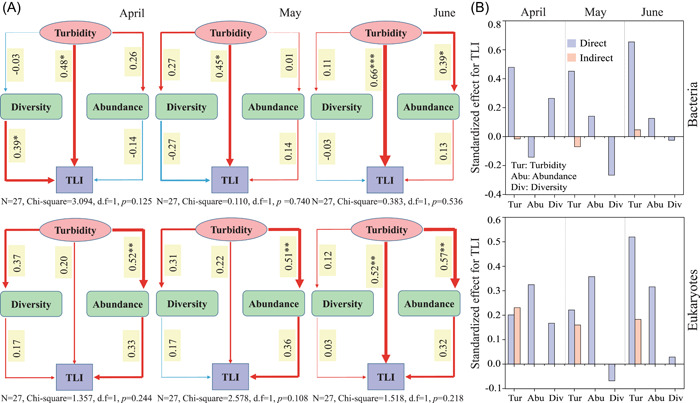
Linkages between abiotic and biotic factors in 3 months. (A) Structural equation models reveal relationships between TLI, turbidity, diversity, and abundance of bacteria and eukaryotes. The width of the arrows represents the strength of the standardized path coefficient. Values above the lines indicate path coefficients between two parameters. The red and blue lines denote positive and negative path coefficients, respectively. Asterisks denote significance (*p < 0.05; **p < 0.01; ***p < 0.001). (B) Direct and indirect effects of abiotic (i.e., turbidity) and biotic (e.g., diversity and abundance) factors on the trophic state (represented by TLI) of the lakes.

Significant correlations were found between TLI and bacterial diversity in April (*p* < 0.05), bacterial abundance in June (*p* < 0.05), and eukaryotic abundance in 3 months (*p* < 0.05 or *p* < 0.001) (Supporting Information: Table S6). Structural equation modeling was used to reveal potential linkages between TLI, turbidity, diversities, and abundances of planktonic bacteria and eukaryotes in 3 months (Figure 3). Turbidity positively affected diversity (except bacteria in April) and abundances of bacteria and eukaryotes, which in turn positively affected TLI (except eukaryotes in April and May) (Figure 3A). Turbidity showed positive effects on TLI, and significant path coefficients were found under most conditions (*p* < 0.05 or *p* < 0.01 or *p* < 0.001). The models displayed good fits to our data (sample number *N* = 27, d.f. = 1), as denoted by the nonsignificant *χ* ^2^ tests in SEM for bacteria (April, *χ* ^2^ = 3.094, *p* = 0.125; May, *χ* ^2^ = 0.110, *p* = 0.740; June, *χ* ^2^ = 0.383, *p* = 0.536) and eukaryotes (April, *χ* ^2^ = 1.357, *p* = 0.244; May, *χ* ^2^ = 2.578, *p* = 0.108; June, *χ* ^2^ = 1.518, *p* = 0.218) (Figure 3A). Turbidity showed both direct and indirect effects on TLI, whereas diversity and abundance of bacteria and eukaryotes only displayed direct effects on TLI (Figure 3B). Consequently, the total effect of turbidity rather than planktonic diversity and abundance showed larger effects on TLI in 3 sampling months.

## DISCUSSION

### Aggravated algal blooms potentially resulting in decreased environmental heterogeneity

Physicochemical properties, Chl‐*α* content, and trophic state changed temporally [22, 32], and many studies prefer to explore influences of individual physicochemical factors (e.g., nitrogen, phosphorus, pH, temperature, salinity, and light) on the water trophic state [5, 33]. However, a comprehensive understanding of the linkage between environmental factors and the water trophic state is rarely reported. Environmental heterogeneity can reflect variations in environmental properties [25], and earlier studies report that water environmental heterogeneity changed temporally in the subtropical Paraná River during flooding [25, 34]. We found that aggravated algal blooms resulted in decreased environmental heterogeneity, which has not been reported before. Previous studies demonstrate that environmental heterogeneity affects ecological processes adjusting microbial community assembly in terrestrial and aquatic ecosystems [25, 35]. Ecological assembly processes determine community structure, species coexistence and abundance, and couple community composition with ecosystem functions [24, 25, 36]. Aggravated algal blooms increased bacterial and eukaryotic diversities and some specific factors (e.g., turbidity) notably affected abundance and diversity of bacteria and eukaryotes. Future studies need to verify this finding in more diverse aquatic ecosystems to allow for better generalization.

### Distribution patterns of bacteria and eukaryotes reveal temporal differences

The community composition of bacteria and eukaryotes in urban lakes changed within a short time, which is similar to findings for other lakes [17, 37, 38]. Taxonomic and phylogenetic similarities of bacterial and eukaryotic communities in the urban lakes decayed with geographical distance, which is consistent with prior findings in other aquatic ecosystems [39, 40]. The significant DDRs were probably attributable to differentiating‐dominated rather than homogenizing‐dominated assembly processes of bacterial and eukaryotic communities. It has been reported that differentiating processes (i.e., dispersal limitation and variable selection) leads to community compositional dissimilarity [41]. The significant differences in the community structure of bacteria and eukaryotes over 3 months might be due to notable shifts in water physicochemical properties. Nutritional (e.g., nitrogen and phosphorus) and nonnutritional abiotic factors (e.g., temperature, turbidity, pH, dissolved oxygen, and salinity) affected community composition of bacteria and/or eukaryotes in the studied and other urban lakes [17, 37, 42, 43]. For different lakes, different environmental factors control planktonic community composition [42, 44]. Generally, microbial community composition is coupled with community function and ecosystem functioning [38], and therefore it is critical to disentangle different factors controlling planktonic community composition throughout time and space.

### Environmental constraints affect bacterial and eukaryotic community assemblies differently

Stochastic and deterministic processes balanced community assembly of bacteria in the studied urban lakes. In the studied urban lakes, stochastic processes dominated eukaryotic community assembly, which is in accordance with a previous notion of eukaryotic community assembly in the Tingjiang River [29] and in pools along the Baltic Sea coast [46]. Yet, this notion differs from earlier studies revealing that both stochastic and deterministic processes balance eukaryotic community assembly in various Chinese lakes and reservoirs [47] as well as in wells in North of Richland of America [48]. Whether stochastic and/or deterministic processes dominate plankton community assembly seems to be affected by environmental heterogeneity and geospatial differences [25, 39]. The selected urban lakes in this study, however, tend to have both a relatively low environmental heterogeneity and geospatial difference, which may lead to the observed differences in bacterial and eukaryotic community assemblies. Furthermore, nutrient‐poor environments trend to have a more determinism‐dominated community assembly, whereas nutrient‐rich environments reveal a more stochasticity‐dominated community assembly [49]. This can be clearly seen for eukaryotic community assembly in the studied urban lakes. Additionally, different ecological processes seem to dominate community assemblies of bacteria versus eukaryotes, which might be also attributable to differences in their lifestyle and cell size [50–52]. Bacteria are typically regarded as unicellular, whereas many eukaryotes are multicellular. For example, hyphomycetes are typically characterized as filamentous fungi, producing extreme branching mycelia [53]. Several eukaryotes (e.g., filamentous fungi and algae) constitute much larger cell sizes than bacteria, and therefore disperse differently in the vegetative state. However, some eukaryotes (e.g., animal, insect, and protozoa) can actively move in water by using feet, tail, and flagella [54], which enables relatively easy access to abundant nutrients and flee from unfavorable conditions (e.g., high‐temperature, low oxygen, high salinity, and predation). Therefore, a previous study has reported that the relative contributions of neutral processes and environmental selection to microbial community assembly directly rely on microbial body size [52]. Environmental constraints of bacteria increased with aggravated algal blooms, whereas environmental constraints of eukaryotes decreased with aggravated algal blooms. Typically, eukaryotes have complex cell structures (e.g., pseudopods) and some eukaryotes occupy relatively high trophic levels in aquatic food webs. In contrast, bacteria occupy relatively low trophic levels in aquatic food webs and live mainly in biofilms [55]. The differences in metabolic activity and dispersal potentials between bacteria and eukaryotes might lead to different changes in environmental constraints of bacteria and eukaryotes during aggravated algal blooms. Additionally, species replacement could also explain the opposite change trend of environmental constraints of bacteria and eukaryotes, suggesting a strong species replacement result in a weak environmental constraint [20].

Coexistence patterns of bacteria and eukaryotes changed temporally, similar to earlier findings on bacterial coexistence pattern across seasons in Lake Taihu [18] and Lake Nanhu [22]. Over time, bacterial and eukaryotic communities showed opposite interaction patterns with decreasing and increasing potential antagonistic interactions, respectively. This pattern occurred in parallel to the opposing effects of environmental constraints on bacteria versus eukaryotes. This phenomenon might imply a close linkage between environmental constraints and potential antagonistic interactions. Some eukaryotes occupy relatively high trophic levels in aquatic food webs [13], therefore, intensive blooms or mass developments might severely intensify competition for food and space. Nutrients (e.g., carbon, nitrogen, and phosphorus sources) can exchange between sediment and water via microbial activities [19], which offset nutrient deficiency for bacterioplankton. Some core species identified from co‐occurrence networks, for example, *Limnohabitans*, *Deinococcus*, and *Acinetobacter*, are important participants of both carbon and phosphorus cycles [56, 57]. From an environmental protection perspective, planktonic interactions should be controlled by changing the presence of core species (e.g., dredging) to weaken community functions for nutrient cycling.

### Biotic and abiotic factors shaping water trophic state

Turbidity rather than diversity and abundance of planktonic bacteria and eukaryotes showed larger effects on water trophic state of the studied lakes, which is opposite to our hypothesis. This phenomenon might be primarily attributable to turbidity affecting the penetrability of light to the water, which in turn affects photosynthesis and biomass of primary producers (e.g., cyanobacteria and green algae) [58, 59]. In addition, turbidity can also affect nutrient availability [60], which in turn influences bacterial and eukaryotic communities. Both bacteria and eukaryotes can consume available nutrients [61], and nutrient limitation increases competition between bacteria and eukaryotes for nutrients and possibly space. Therefore, nutrient limitation affects abundance, community composition, and the function of plankton [19, 42]. Though nutrients can be released from sediments into the water [11, 19], this supplement might not meet the demand for large populations of bacteria and eukaryotes at short time scales. Considering multiple copies of bacterial 16S rRNA gene and eukaryotic 18S rRNA gene, the limitation of quantitative polyerase chain reaction (qPCR) might affect the linkage between water trophic state and abundance of bacteria and eukaryotes. Future studies will precisely estimate the relationship between planktonic abundance and water trophic state by accurately quantifying planktonic abundance by using novel techniques (e.g., time of flight mass spectrometer). Understanding biotic and abiotic factors affecting the water trophic state is important to formulate efficient environmental protection policies. From the viewpoint of practical lake management, environmental protection measures (e.g., dredging and lanthanum‐modified bentonite) should be jointly used during the bloom development stage to mitigate or inhibit algal bloom damage.

## CONCLUSION

Collectively, our sequencing datasets and statistical analyses are the first to reveal that aggravated algal blooms decreased the environmental heterogeneity of urban lakes. Planktonic bacteria and eukaryotes displayed differentiation strategies in response to aggravated algal blooms, with embodiments of opposite changes in species replacement, richness difference, environmental constraint, and potential antagonistic interaction over time. Turbidity shows significant effects on abundance and diversity of bacteria and eukaryotes. Consequently, turbidity rather than diversity and abundance of planktonic bacteria and eukaryotes control more on water trophic state. Our findings extend our current understanding of plankton's response to aggravated algal bloom and planktonic diversity maintenance mechanisms. These ecological findings will enable and guide the formulation of environmental protection policies to better and more efficiently mitigate the tropical state of lakes. Future studies need to verify these findings in more different lakes over a long time scale.

## METHODS

### Water sample collection and physicochemical property

In each month (i.e., April, May, and June) in 2021, we collected 27 surface (0–0.3 m) water samples from representative sites in 12 shallow lakes in Wuhan, China (Supporting Information: Table S1 and Figure S1). A total of 81 water samples were collected by using a Wanzun Water Sampler, and water samples were stored in sterile polypropylene water bags and immediately kept at approximately 4°C in a portable refrigerator. The visible objects (e.g., leaves and small animals) were removed via filtering through 5 mm sterile gauzes. Approximately 100 ml of the filtered water samples were used to determine water physicochemical properties, and the remaining part was then filtered through 0.22‐μm polycarbonate membranes (Millipore Corporation) for molecular analyses.

Water temperature, pH, dissolved oxygen, turbidity, and electrical conductivity were determined in situ with a portable YSI Pro1020 Water Quality Tester (Visal). We measured concentrations of total phosphorus, soluble reactive phosphorus, total nitrogen, ammonia nitrogen, nitrate nitrogen, chemical oxygen demand, calcium, magnesium, iron, and Chl‐*α* according to standard approaches and protocols. Detailed descriptions of the determination of water physicochemical properties are summarized in supporting information (Supporting Information Method 1).

The key parameters of Chl‐*α*, total nitrogen, total phosphorus, and chemical oxygen demand were used as evaluation factors to calculate the TLI of urban lakes, and detailed algorithms have been reported previously [8] and also summarized in supplementary materials (Supporting Information Method 1). Environmental heterogeneity was calculated based on physicochemical factor dissimilarity [25], and detailed descriptions are summarized in supporting information (Supporting Information Method 1).

### Molecular analyses

Genomic DNA of plankton was extracted with a PowerWater Isolation Kit (MOBIO) using the manufacturer's instructions. DNA purity and concentration were measured with a NanoDrop 2000 Spectrophotometer (Thermo Fisher Scientific). All DNA samples were placed at −80°C.

Universal primers 338F (5′‐ACT CCT ACG GGA GGC AGC A‐3′) and 806R (5′‐GGA CTA CHV GGG TWT CTA AT‐3′) were used to partially amplify the bacterial 16 S rRNA gene targeting the V3‐V4 region [62]. Universal primers Eu565F (5′‐CCA GCA SCY GCG GTA ATT CC‐3′) and Eu981R (5′‐ACT TTC GTT CTT GAT YRA TGA‐3′) were used to partially amplify eukaryotic 18S rRNA gene targeting the V4 region [63]. Quantitative PCR was used to determine the absolute abundances of bacteria and eukaryotes using an ABI VIIA 7 Cycle Real‐time PCR System (Applied Biosystems). The amplicon sequencing was conducted on an Illumina MiSeq platform at Personal Biotechnology Co., Ltd. The raw sequences were run through the QIIME2 pipeline and DADA2 to obtain denoised, chimera‐free, and nonsingleton ASVs [64]. For subsequent analyses, sequencing effort was done based on a minimum number of sample sequences and we eliminated all ASVs that contained < 20 reads. The phylogenetic trees of bacteria and eukaryotes were built based on gene sequences by using the FastTree tool [65].

### Data analysis

One‐way analysis of variance was used to reflect significant differences in the data. Venn diagram, NMDS plot, and ANOSIM were applied to estimate planktonic compositional variations to reveal general distribution patterns of plankton. We calculated the taxonomic distances represented by Bray–Curtis dissimilarity and phylogenetic distances represented by beta mean nearest taxon distance (βMNTD) of both bacterial and eukaryotic communities. Additionally, we evaluated DDRs between geographical distance and community similarities based on taxonomic (Bray–Curtis similarity) and phylogenetic (1‐βMNTD) analyses. The RDA was used to elucidate the effects of physicochemical factors on bacterial and eukaryotic community composition. We assessed the pure effect (without other factor's influence) of each tested physicochemical parameter on community composition of planktonic bacteria and eukaryotes using PERMAVONA [37]. To investigate the effects of species replacement and richness difference on community composition of bacteria and eukaryotes, we disassembled taxonomic β‐diversity based on Bray–Curtis dissimilarity by using “agricolae” and “adespatial” packages, and visualized by using “ggtern” and “vcd” packages of R [20].

Mantel correlograms were constructed to test whether significant phylogenetic signals of species occur at short of phylogenetic distances along environmental gradients by using the “mantel.correlog” function in the “vegan” package [48]. Null model, neutral model, and niche breadth analysis were employed to estimate community assemblies of planktonic bacteria and eukaryotes [29, 66, 67]. According to divergences in taxonomic (BrayCurtis‐based Raup‐Crick [RC_bray_]) and phylogenetic (β‐nearest taxon index [βNTI]) diversities, the null model was applied to compute proportions of stochastic and deterministic processes by applying the “picante” package. Ecological assembly processes were distinguished according to RC_bray_ and βNTI, including homogeneous selection (βNTI < −2), variable selection (βNTI > 2), dispersal limitation (| βNTI | < 2 and RC_bray_ > 0.95), homogenizing dispersal (| βNTI | < 2 and RC_bray_ < −0.95), “undominated” processes (| βNTI | < 2 and | RC_bray_ < 0.95), deterministic processes (| βNTI |  > 2), stochastic processes (| βNTI | < 2), homogenizing processes (homogenizing dispersal plus homogeneous selection), and differentiating processes (variable selection plus dispersal limitation). Detailed descriptions of these ecological assembly processes are reported previously [66, 68]. We computed the Levins' niche breadth index of “*B*” and community‐level *B* value (*Bcom*) to infer the niche breath by applying the “spaa” package in R, whereby large *Bcom* values reflect good metabolic flexibility [69]. Sloan neutral model was applied to estimate the contribution of stochasticity to community assembly via evaluating the migration rate as “*m*” value [70].

The ASVs observed in > 50% of samples in each month (>14 samples) were used to build co‐occurrence networks [22]. Co‐occurrence networks were visualized by using the software Gephi v. 0.9.2 (https://gephi.org/) with a significance *p* < 0.01 and Spearman's correlation coefficients (*r*) > 0.6 [22]. Structural equation modeling (SEM) was built using “plspm” and “sem” package of R.

## AUTHOR CONTRIBUTIONS

**Wenjie Wan**: Conceptualization, methodology, writing–original draft, validation, investigation, and funding acquisition. **Hans‐Peter Grossart**: Writing–review and editing. **Donglan He**: Conceptualization, methodology, and validation. **Wenzhi Liu**: Conceptualization and methodology. **Shuai Wang**: methodology and software. **Yuyi Yang**: Conceptualization, writing–review and editing, and funding acquisition.

## CONFLICT OF INTEREST STATEMENT

The authors declare no conflict of interest.

## Supporting information

Supporting information.

## Data Availability

The sequencing data sets were deposited in the database of the National Center for Biotechnology Information Short Read Archive database under accession numbers PRJNA818785 (bacteria) and PRJNA818786 (eukaryotes). Important R codes (published by statisticians) are summarized in Supporting Information. Supplementary materials (figures, tables, scripts, graphical abstract, slides, videos, Chinese translated version, and updated materials) may be found in the online DOI or iMeta Science https://www.imeta.science/.
