# Supplementary material for: Differentiation strategies for planktonic bacteria and eukaryotes in response to aggravated algal blooms in urban lakes
Source: Imeta. 2023 Jan 31;2(1):e84. doi: 10.1002/imt2.84 (PMC10989909; doi:10.1002/imt2.84)
Supplement: Supplementary file 1 — Supporting information. [file IMT2-2-e84-s001.docx]

Supporting information for:

**Differentiation strategies of planktonic bacteria and eukaryotes in response to aggravated algal blooms of urban lakes**

**Running title**: **Responses of plankton to lake algal blooms**

Wenjie Wan^1, 5^, Hans-Peter Grossart^3, 4^, Donglan He^2^, Wenzhi Liu^1, 5^, Shuai Wang^2^, Yuyi Yang^1, 5, *^

^1^Key Laboratory of Aquatic Botany and Watershed Ecology Wuhan Botanical Garden, Chinese Academy of Sciences, Wuhan 430074, PR China

^2^College of Life Science, South-Central Minzu University, Wuhan 430070, PR China

^3^Leibniz-Institute for Freshwater Ecology and Inland Fisheries (IGB), Dept. of Plankton and Microbial Ecology, 16775, Neuglobsow, Germany

^4^University of Potsdam, Institute of Biochemistry and Biology, Maulbeerallee 2, 14469, Potsdam, Germany

^5^Danjiangkou Wetland Ecosystem Field Scientific Observation and Research Station, Chinese Academy of Sciences & Hubei Province, Wuhan 430074, PR China

*Corresponding Author: [yangyy@wbgcas.cn](mailto:yangyy@wbgcas.cn) (Yuyi Yang)

**ORCID**: Yuyi Yang: <https://orcid.org/0000-0001-9807-6844>

Wenjie Wan: https://orcid.org/0000-0001-7150-6138

Address: Wuhan Botanical Garden, Lumo Road No.1, Wuchang District, Wuhan, PR China. Phone: +86-27-87700853; Fax: +86-27-87510251.

**Supplementary Method 1**: Determination of water physicochemical properties and Description of environmental heterogeneity

Water total phosphorus (TP) was digested using K_2_S_2_O_8_ at 121°C for 30 min, and the content of TP and soluble reactive phosphorus (SRP) was determined using ammonium molybdate spectrometric method. Water total nitrogen (TN) was digested using alkaline K_2_S_2_O_8_ at 121°C for 30 min, and then cooled before 10% hydrochloric acid of 1 mL was added. The content of TN was determined using spectrophotometric method at 275 nm and 220 nm. Water nitrate nitrogen (NO_3_^–^–N) was processed by using 10% hydrochloric acid, and was determined by using pectrophotometric method at 275 nm and 220 nm. Water ammonia nitrogen (NH_4_^+^–N) was determined by using sodium nitroferricyanide assay. Chemical oxygen demand (COD) was determined by using potassium dichromate assay. Calcium (Ca), magnesium (Mg), and iron (Fe) was determined using an AA240FS atomic-absorption spectrophotometer (Varian Company, USA). The content of chlorophyll-*α* (Chl-*α*) was estimated by using alcohol spectrometric method.

The Chl-α, TN, TP, and COD were used for quantitative evaluation of trophic level for eutrophication (Zhang et al., 2011), as measured by trophic level index (TLI):


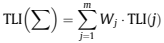


where TLI(∑) is integrated trophic level index; TLI(j) is trophic level index of j, Wj is correlative weighted score for trophic level index of j.


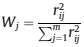


where Wj is correlative weighted score for trophic level index of j; Rij is relative coefficient (for Chl-α is 1, for TN is 0.82, for TP is 0.84, and for COD is 0.83).

TLI (Chl-α) = 10$\times$(2.5 + 1.086$\times$lnChl-α) (1)

TLI (TN) = 10$\times$(5.453 + 1.694$\times$lnTN) (2)

TLI (TP) = 10$\times$(9.436 + 1.624$\times$lnTP) (3)

TLI (COD) = 10$\times$(0.109 + 2.66$\times$lnCOD) (4)

The lake trophic state was further classified based on the TLI(∑) value as either oligotrophic (TLI(∑) < 30), mesotrophic (30 < TLI(∑) < 50), eutrophic (50 < TLI(∑) < 60), middle eutrophic (60< TLI(∑) < 70), or hypereutrophic (TLI(∑) > 70).

Environmental heterogeneity was estimated by computing the average dissimilarity between sites (Ed) (Huber et al., 2020) based on 14 abiotic variables (i.e., temperature, pH, turbidity, dissolved oxygen, electrical conductivity, TP, SRP, TN, NH_4_^+^–N, NO_3_^–^–N, COD, Ca, Mg, and Fe). The Ed was calculated based on a Euclidean distance by using the following equation, Ed = Euc/Euc_max_ + 0.001. where Euc is the Euclidean distance between two sites and Euc_max_ corresponds to the maximum Euclidean distance

considering all the pairwise distances in the overall dataset. 0.001 was added to account for zero similarity between sites.

**REFERENCES**

Huper, Paula, Sebastian Metz, U Fernando Unrein, Gisela Mayora, Hugo Sarmento, and Melina Devercelli. 2020. “”Environmental heterogeneity determines the ecological processes that govern bacterial metacommunity assembly in a floodplain river system.” *The ISME Journal* 14: 2951–2966.

https://doi.org/10.1038/s41396-020-0723-2

Zhang, Jianying, Wanmin Ni, Yang Luo, R. Jan Stevenson, and Jiaguo Qi. 2011. “Response of freshwater algae to water quality in Qinshan Lake within Taihu Watershed, China.” *Physics and Chemistry of the Earth* 36, 360–365. https://doi.org/10.1016/j.pce.2010.04.018

**Table S1** Information of sampling sites covering 12 urban lakes in Wuhan City.

| Lake Name | Lake abbreviation | Waters from each lake | Sample number | Longitude (°/E) | Latitude (°/N) |
| --- | --- | --- | --- | --- | --- |
| Lake Nanhu | NH | 7 | S1, S2, S3, S4, S5, S6, S7 | 114.385706 | 30.490831 |
| Lake Yezhihu | YZ | 2 | S8, S9 | 114.331419 | 30.465058 |
| Lake Tangxunhu | TX | 3 | S10, S11, S12 | 114.328003 | 30.414783 |
| Lake Huangjiahu | HJ | 2 | S13, S14 | 114.280700 | 30.432183 |
| Lake Qinglinghu | QL | 2 | S15, S16 | 114.247864 | 30.440392 |
| Lake Tanghu | TH | 1 | S17 | 114.154203 | 30.472647 |
| Lake Wanjiahu | WJ | 1 | S18 | 114.175833 | 30.469311 |
| Lake Yuehu | YH | 2 | S19, S20 | 114.257800 | 30.557575 |
| Lake Huanzihu | HZ | 1 | S21 | 114.277725 | 30.602414 |
| Lake Shahu | SH | 2 | S22, S23 | 114.339464 | 30.575211 |
| Lake Ziyanghu | ZY | 2 | S24 | 114.299936 | 30.535319 |
| Lake Donghu | DH | 3 | S25, S26, S27 | 114.369719 | 30.577592 |

**Table S2** Pearson’s correlations between physicochemical factors and Chl-*α* content and TLI. Asterisks denote significance (*, *p* < 0.05; **, *p* < 0.01; ***, *p* < 0.001).

| Factor | Chl-*α* content | | | TLI | | |
| --- | --- | --- | --- | --- | --- | --- |
|  | April | May | June | April | May | June |
| Temp | 0.200 | -0.723*** | -0.263 | 0.496** | -0.392* | -0.414* |
| pH | -0.021 | 0.108 | -0.208 | 0.072 | -0.117 | -0.241 |
| Tur | 0.710*** | 0.530** | 0.473* | 0.427* | 0.384* | 0.702*** |
| DO | 0.366 | 0.210 | 0.572** | -0.434* | 0.020 | 0.321 |
| EC | -0.361 | -0.155 | 0.560** | -0.007 | 0.402* | 0.642*** |
| TP | 0.013 | 0.338 | 0.429* | 0.719*** | 0.776*** | 0.668*** |
| SRP | -0.263 | -0.093 | 0.222 | 0.497** | 0.400* | 0.510** |
| TN | -0.147 | -0.068 | 0.505** | 0.625*** | 0.607*** | 0.642** |
| NH4 | -0.240 | -0.134 | 0.322 | 0.580** | 0.547** | 0.536** |
| NO3 | 0.156 | -0.139 | 0.388* | 0.291 | 0.225 | 0.126 |
| COD | 0.129 | 0.349 | 0.595** | 0.747*** | 0.795*** | 0.851*** |
| Ca | 0.087 | 0.338 | 0.564** | 0.740*** | 0.624*** | 0.666*** |
| Mg | 0.012 | -0.018 | 0.616*** | 0.414* | 0.402* | 0.684*** |
| Fe | 0.153 | 0.407* | 0.327 | 0.680*** | 0.785*** | 0.554* |

Abbreviations: Chl-*α*, Chlorophyll-*α;* TLI, trophic level index; Temp, temperature; DO, dissolved oxygen; Tur, turbidity; EC, electrical conductivity; TP, total phosphorus; SRP, soluble reactive phosphorus; TN, total nitrogen; NH_4_^+^–N, ammonia nitrogen; NO_3_^–^–N, nitrate nitrogen; COD, chemical oxygen demand; Ca, calcium; Mg, magnesium; and Fe, iron.

**Table S3** Pearson’s correlations between physicochemical variables and absolute abundances of bacteria and eukaryotes in each sampling month. Asterisks denote significance (*, *p* < 0.05; **, *p* < 0.01; ***, *p* < 0.001).

| Factor | Bacterial abundance | | | Eukaryotic abundance | | |
| --- | --- | --- | --- | --- | --- | --- |
|  | April | May | June | April | May | June |
| Temp | -0.213 | -0.168 | -0.158 | 0.116 | -0.748*** | -0.295 |
| pH | 0.133 | 0.540** | 0.006 | 0.027 | 0.076 | -0.007 |
| Tur | 0.434* | 0.006 | 0.393* | 0.518** | 0.506** | 0.567** |
| DO | 0.463* | 0.618*** | 0.521** | 0.122 | 0.004 | 0.403* |
| EC | -0.096 | -0.470* | 0.403* | -0.101 | -0.239 | 0.572** |
| TP | -0.171 | 0.110 | 0.238 | 0.019 | 0.169 | 0.405* |
| SRP | -0.281 | -0.029 | -0.187 | -0.207 | -0.181 | 0.052 |
| TN | -0.353 | -0.184 | 0.184 | -0.064 | -0.182 | 0.312 |
| NH4 | -0.333 | -0.196 | 0.107 | -0.149 | -0.239 | 0.217 |
| NO3 | -0.354 | -0.492** | -0.060 | 0.267 | -0.107 | -0.129 |
| COD | -0.091 | -0.105 | 0.336 | 0.186 | 0.203 | 0.486* |
| Ca | -0.271 | 0.054 | 0.467* | 0.098 | 0.143 | 0.530** |
| Mg | -0.039 | 0.063 | 0.222 | -0.021 | -0.243 | 0.367 |
| Fe | -0.119 | -0.020 | 0.148 | 0.228 | 0.201 | 0.299 |

Abbreviations: Temp, temperature; DO, dissolved oxygen; Tur, turbidity; EC, electrical conductivity; TP, total phosphorus; SRP, soluble reactive phosphorus; TN, total nitrogen; NH_4_^+^–N, ammonia nitrogen; NO_3_^–^–N, nitrate nitrogen; COD, chemical oxygen demand; Ca, calcium; Mg, magnesium; and Fe, iron.

**Table S4** Pearson’s correlations between relative abundances of plankton and Chl-*α* and TLI in different sampling months. Asterisks denote significance (*, *p* < 0.05; **, *p* < 0.01; ***, *p* < 0.001).

| Parameters | Whole period | | April | | May | | June | |
| --- | --- | --- | --- | --- | --- | --- | --- | --- |
|  | Chl-*α* | TLI | Chl-*α* | TLI | Chl-*α* | TLI | Chl-*α* | TLI |
| Proteobacteria | -0.275* | -0.207 | -0.161 | 0.118 | -0.343 | -0.437* | -0.067 | -0.383* |
| Actinobacteria | 0.165 | 0.232* | 0.238 | 0.063 | 0.019 | 0.260 | 0.090 | 0.383* |
| Bacteroidetes | -0.045 | -0.210 | -0.385* | -0.428* | 0.189 | 0.349 | -0.179 | -0.029 |
| Firmicutes | -0.051 | 0.029 | -0.045 | -0.154 | 0.249 | 0.098 | -0.039 | 0.048 |
| Deinococcus-Thermus | -0.039 | 0.008 | 0.297 | -0.006 | -0.175 | -0.209 | -0.305 | -0.295 |
| Cyanobacteria | 0.242* | 0.074 | 0.373* | -0.146 | 0.641*** | 0.347 | 0.407* | -0.038 |
| Verrucomicrobia | 0.307** | 0.144 | 0.414* | 0.027 | 0.603*** | 0.179 | 0.333 | 0.162 |
|  |  |  |  |  |  |  |  |  |
| Chlorophyta | 0.332** | 0.471*** | 0.297 | 0.618*** | 0.194 | 0.457* | 0.418* | 0.477* |
| Rotifera | 0.184 | 0.345** | 0.080 | 0.081 | 0.279 | 0.455* | 0.189 | 0.540** |
| Arthropoda | -0.231* | -0.358*** | -0.365 | -0.424* | -0.143 | -0.315 | -0.305 | -0.344 |
| Chytridiomycota | 0.081 | 0.180 | 0.338 | 0.243 | 0.606*** | 0.374 | -0.102 | 0.190 |
| Dinophyceae | 0.023 | -0.098 | 0.502** | 0.212 | 0.439* | 0.103 | -0.313 | -0.385* |
| Chrysophyceae | -0.253* | -0.235* | -0.333 | -0.038 | -0.299 | -0.252 | -0.298 | -0.356 |
| Bacillariophyta | 0.085 | -0.077 | -0.094 | 0.031 | -0.100 | -0.292 | 0.145 | 0.171 |
| Streptophyta | -0.067 | -0.129 | 0.065 | 0.242 | -0.119 | -0.200 | -0.037 | 0.060 |

**Table S5** Topological parameters of co-occurrence networks for bacteria and eukaryotes in the three sampling months.

| Parameters | Bacteria | | | Eukaryotes | | | Plankton (Bacteria + Eukaryotes) | | |
| --- | --- | --- | --- | --- | --- | --- | --- | --- | --- |
|  | April | May | June | April | May | June | April | May | June |
| Node | 291 | 341 | 426 | 63 | 103 | 109 | 361 | 431 | 540 |
| Edge | 7718 | 3822 | 8444 | 670 | 1322 | 1236 | 9064 | 4662 | 9258 |
| Positive edge | 6954 | 3438 | 8100 | 668 | 1318 | 1184 | 8274 | 4296 | 8768 |
| Negative edge | 764 | 384 | 344 | 2 | 4 | 52 | 790 | 366 | 490 |
| Positive edge / Negative edge | 9.10 | 14.95 | 23.55 | 334 | 329.5 | 22.77 | 10.47342 | 11.7377 | 17.89388 |
| Average degree | 26.522 | 11.208 | 19.822 | 10.635 | 12.835 | 11.339 | 25.108 | 10.817 | 17.144 |
| Diameter | 7 | 9 | 10 | 5 | 8 | 8 | 9 | 11 | 10 |
| Graph density | 0.091 | 0.033 | 0.047 | 0.172 | 0.126 | 0.105 | 0.07 | 0.025 | 0.032 |
| modularity | 0.631(8) | 0.591(13) | 0.454(11) | 0.452(4) | 0.555(5) | 0.425(9) | 0.632(8) | 0.656(15) | 0.541(14) |
| Average clustering coefficient | 0.633 | 0.432 | 0.462 | 0.634 | 0.657 | 0.613 | 0.59 | 0.469 | 0.472 |
| Average path length | 2.867 | 3.682 | 3.285 | 2.459 | 3.095 | 3.278 | 3.091 | 4.024 | 3.561 |

**Table S6** Pearson’s correlations between TLI and abundance and diversity of bacteria and eukaryotes in each sampling month. Asterisks denote significance (*, *p* < 0.05; **, *p* < 0.01; ***, *p* < 0.001).

| Month | Bacterial community | | Eukaryotic community | |
| --- | --- | --- | --- | --- |
|  | diversity | abundance | diversity | abundance |
| April | 0.401* | -0.111 | 0.360 | 0.486* |
| May | -0.138 | 0.127 | 0.148 | 0.445* |
| June | 0.036 | 0.385* | 0.176 | 0.617*** |


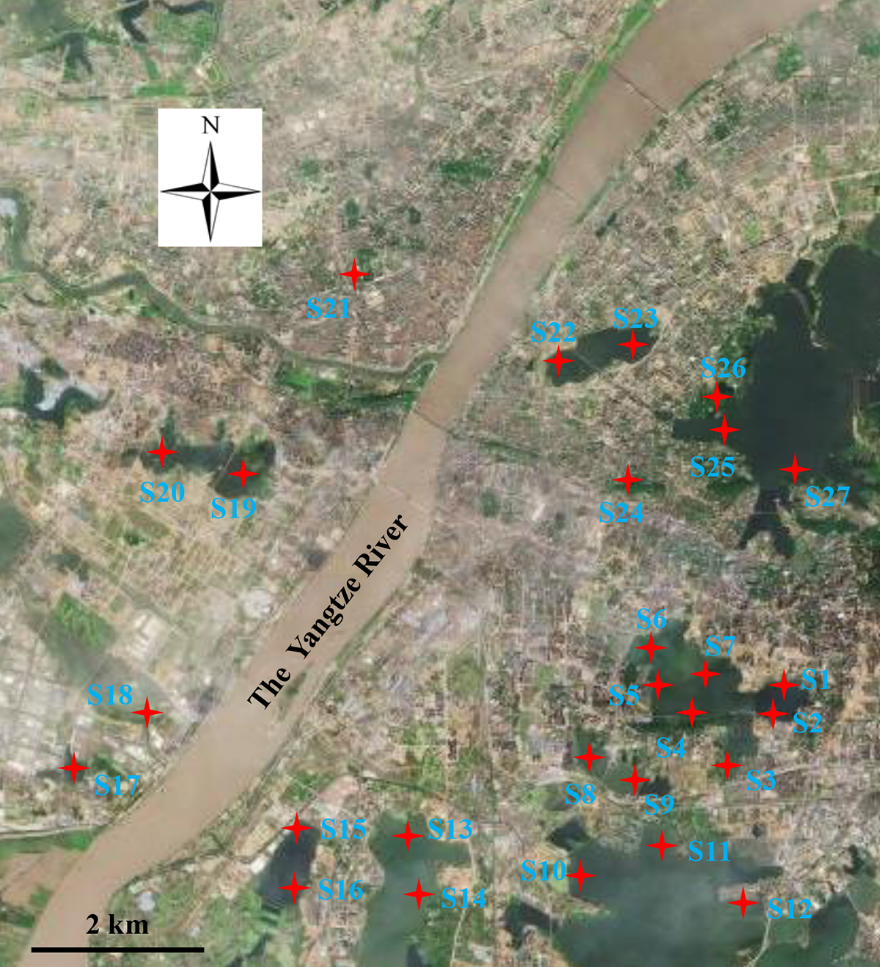


**Figure S1**. The sampling sites in Wuhan lakes.


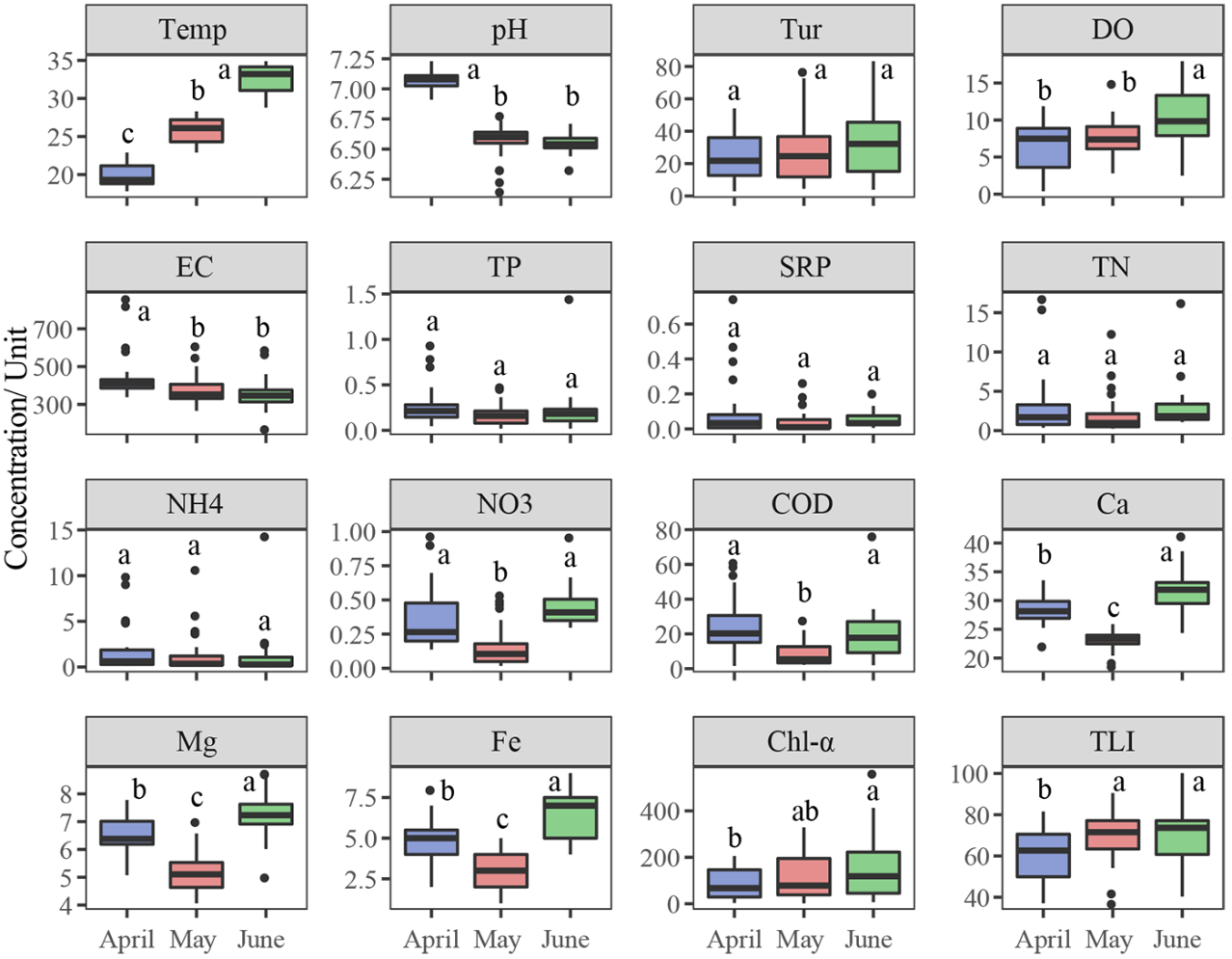


**Figure S2**. Box plots showing differences in physicochemical properties and trophic level in April, May, and June. Parameter units are: °C (Temp), NTU (Tur), mg/L (DO, TP, SRP, TN, NH_4_^+^–N, NO_3_^–^–N, COD, Ca and Mg), μs/cm (EC), and μg/L (Fe and Chl-*α*). Abbreviations: Temp, temperature; DO, dissolved oxygen; Tur, turbidity; EC, electrical conductivity; TP, total phosphorus; SRP, soluble reactive phosphorus; TN, total nitrogen; NH_4_^+^–N, ammonia nitrogen; NO_3_^–^–N, nitrate nitrogen; COD, chemical oxygen demand; Ca, calcium; Mg, magnesium; Fe, iron; Chl-*α*, chlorophyll-*α*; and TLI, trophic level index. Letters above the columns denote significance (*p* < 0.05).


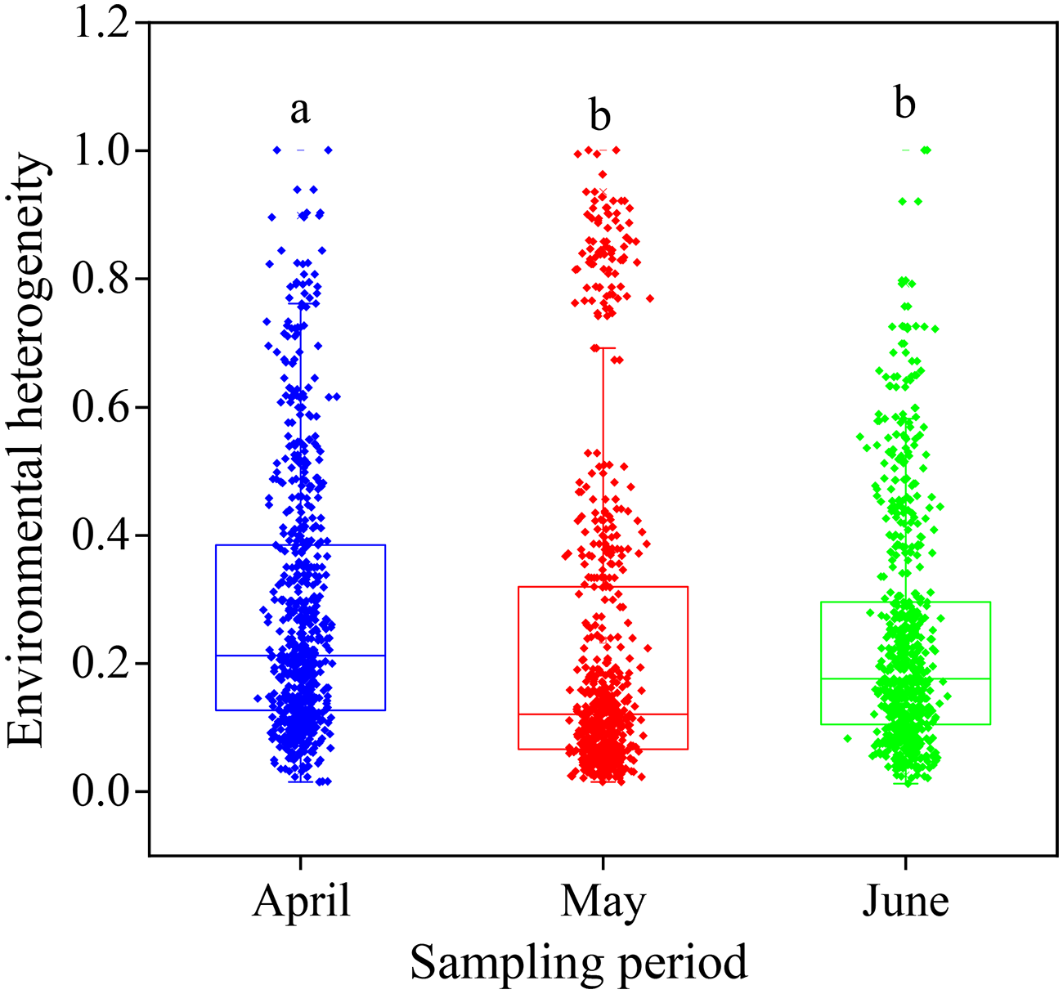


**Figure S3**. Difference in environmental heterogeneity in three sampling months (i.e., April, May, and June). Different letters above columns represent significance (*p* < 0.05).


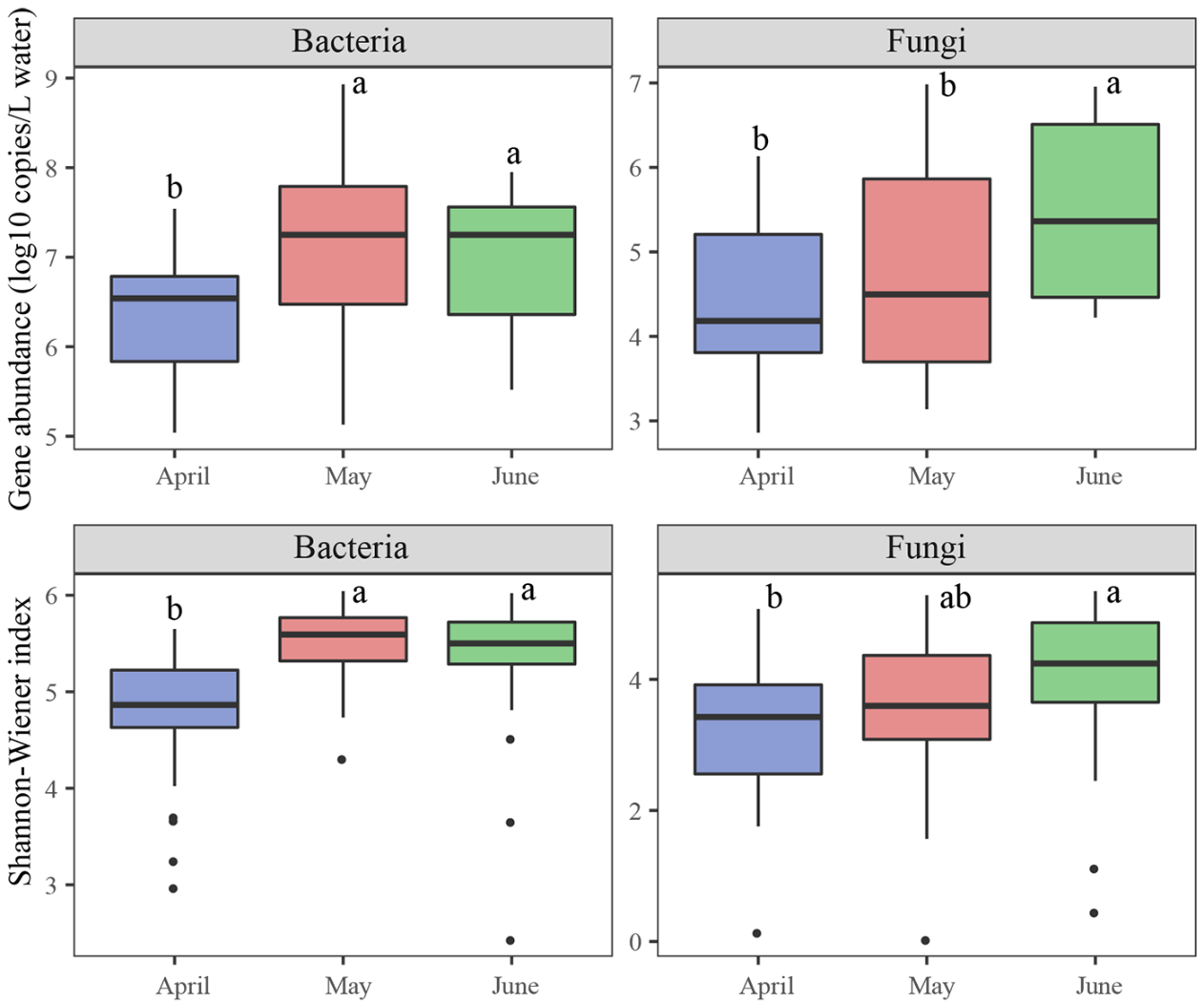


**Figure S4**. Differences in absolute abundances and community diversity (represented by Shannon-Wiener index) and of bacteria and eukaryotes among three sampling months. Different letters above the column denote significance (*p* < 0.05).


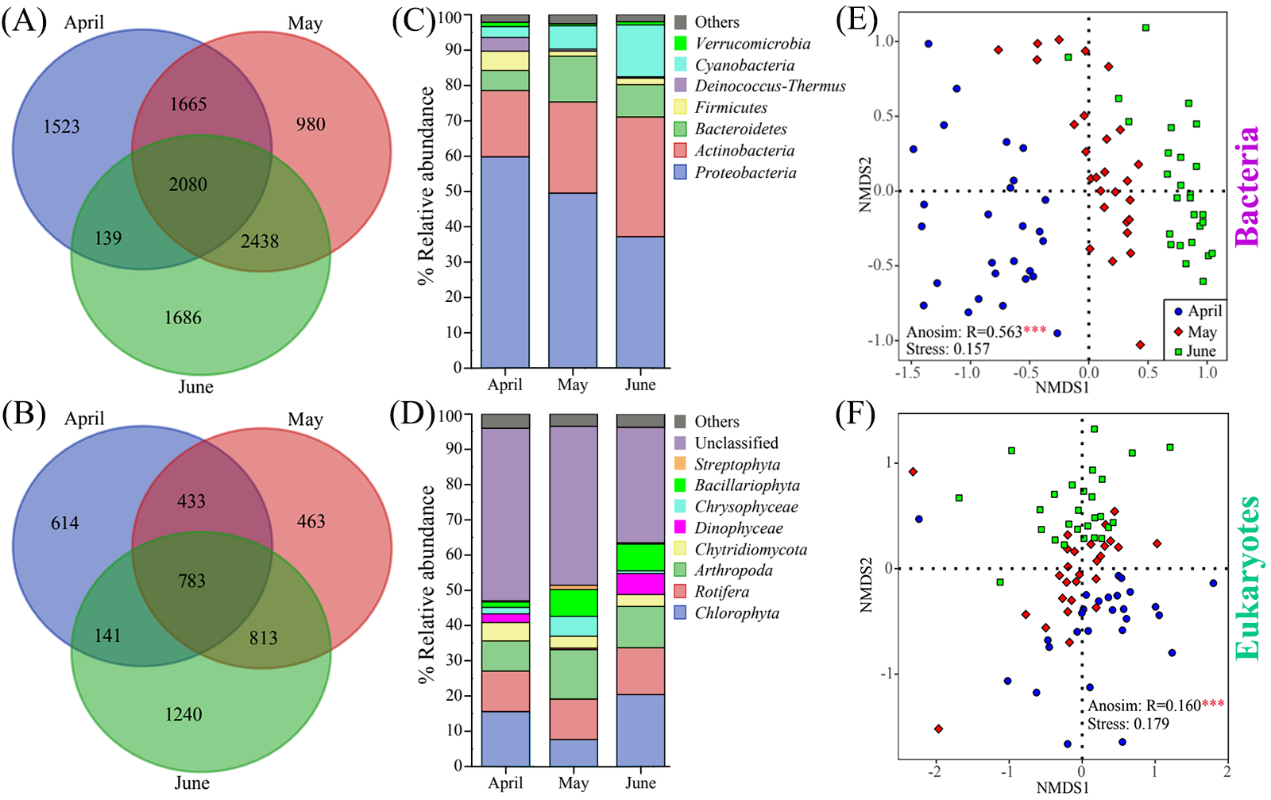


**Figure S5**. Community composition of bacteria and eukaryotes in April, May, and June. Venn diagrams show shared species of bacterial (A) and eukaryotic (B) communities in the three months. Stacked plots reflect relative abundances (> 1%) of bacterial (C) and eukaryotic (D) phyla-classes. Nonmetric multidimensional scaling plots display compositional variations in bacterial (E) and eukaryotic (F) communities among three months. Asterisks in (E) and (F) denote significance (***, *p* < 0.001).


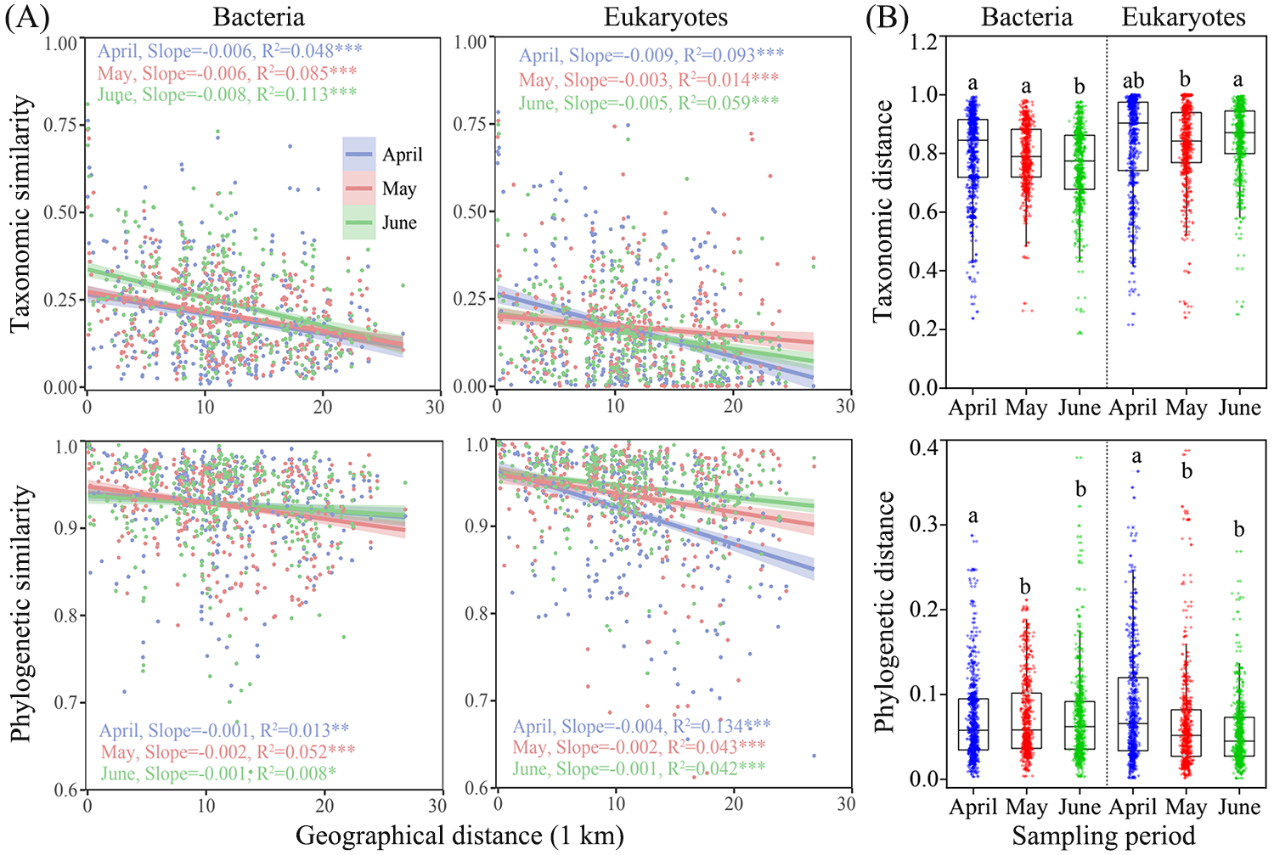


**Figure S6**. Taxonomic and phylogenetic β-diversities of bacterial and eukaryotic communities in lake water in three months. (A) Linear regressions show distance-decay relationships between geographical distance and taxonomic and phylogenetic similarity of bacterial and eukaryotic communities. Asterisks denote significance (*, *p* < 0.05; **, *p* < 0.01; ***, *p* < 0.001). (B) Box plots display differences in taxonomic and phylogenetic distances of bacterial and eukaryotic communities in three months. Different letters above columns represent significance (*p* < 0.05).


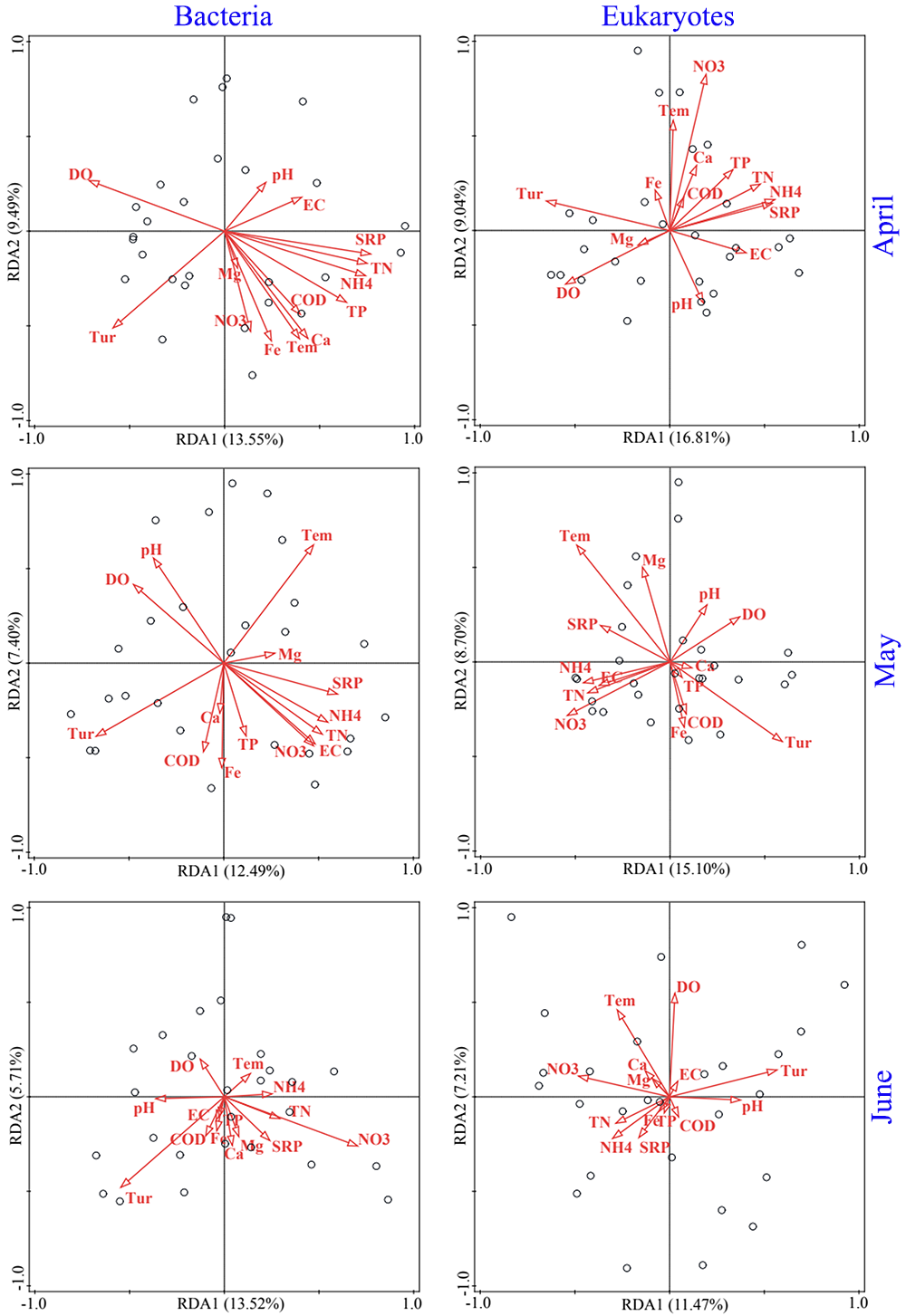


**Figure S7**. Redundancy analysis showing effects of tested physicochemical factors on community compositions of bacteria and eukaryotes in three months. Abbreviations: Temp, temperature; DO, dissolved oxygen; Tur, turbidity; EC, electrical conductivity; TP, total phosphorus; SRP, soluble reactive phosphorus; TN, total nitrogen; NH_4_^+^–N, ammonia nitrogen; NO_3_^–^–N, nitrate nitrogen; COD, chemical oxygen demand; Ca, calcium; Mg, magnesium; and Fe, iron.


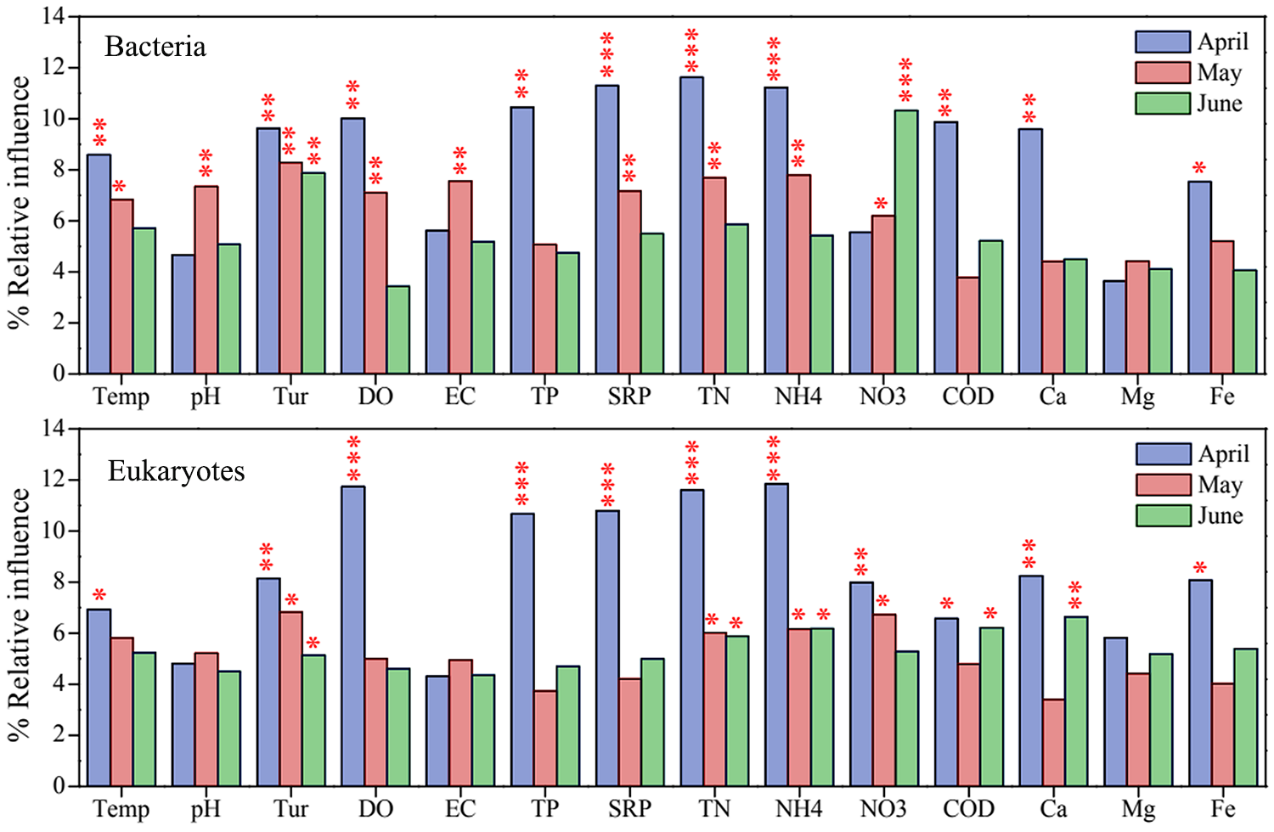


**Figure S8**. Permutational multivariate analysis of variance showing pure effect of each tested physicochemical parameter on bacterial and eukaryotic community compositions in different months. Asterisks denote significance (*, *p* < 0.05; **, *p* < 0.01; ***, *p* < 0.001). Abbreviations: Temp, temperature; DO, dissolved oxygen; Tur, turbidity; EC, electrical conductivity; TP, total phosphorus; SRP, soluble reactive phosphorus; TN, total nitrogen; NH_4_^+^–N, ammonia nitrogen; NO_3_^–^–N, nitrate nitrogen; COD, chemical oxygen demand; Ca, calcium; Mg, magnesium; and Fe, iron.


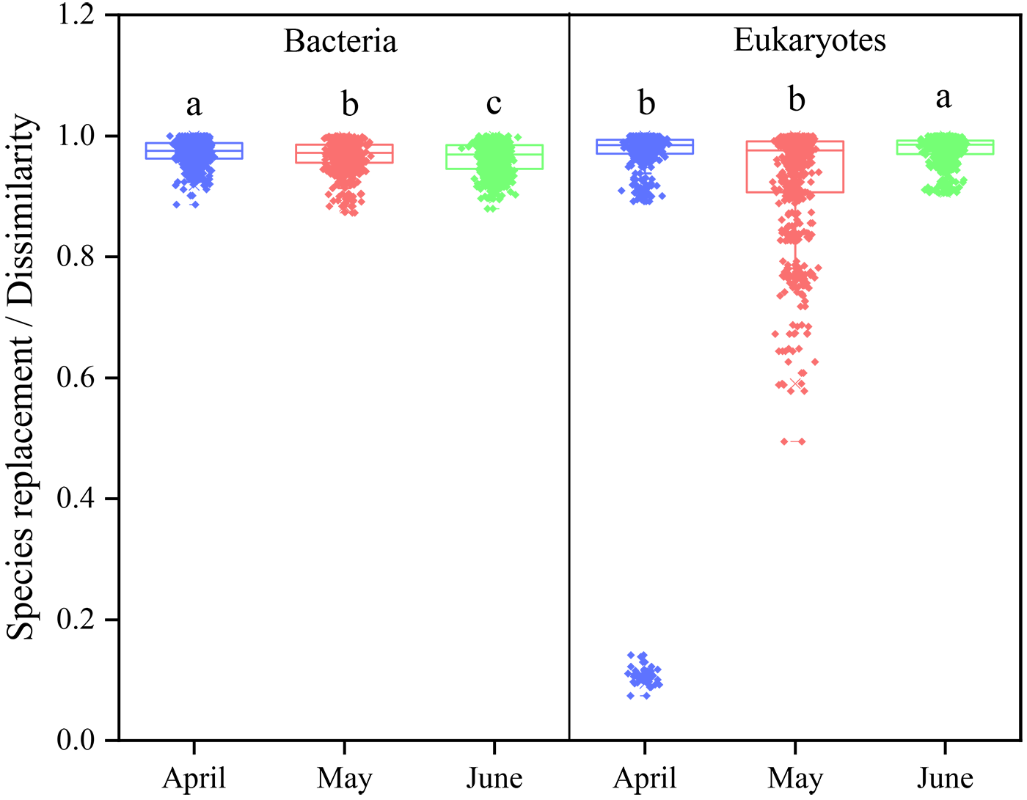


**Figure S9**. Differences in species replacement/dissimilarity of bacterial and eukaryotic communities among three sampling months. Different lowercae letters above columns denote significance (*p* < 0.05).


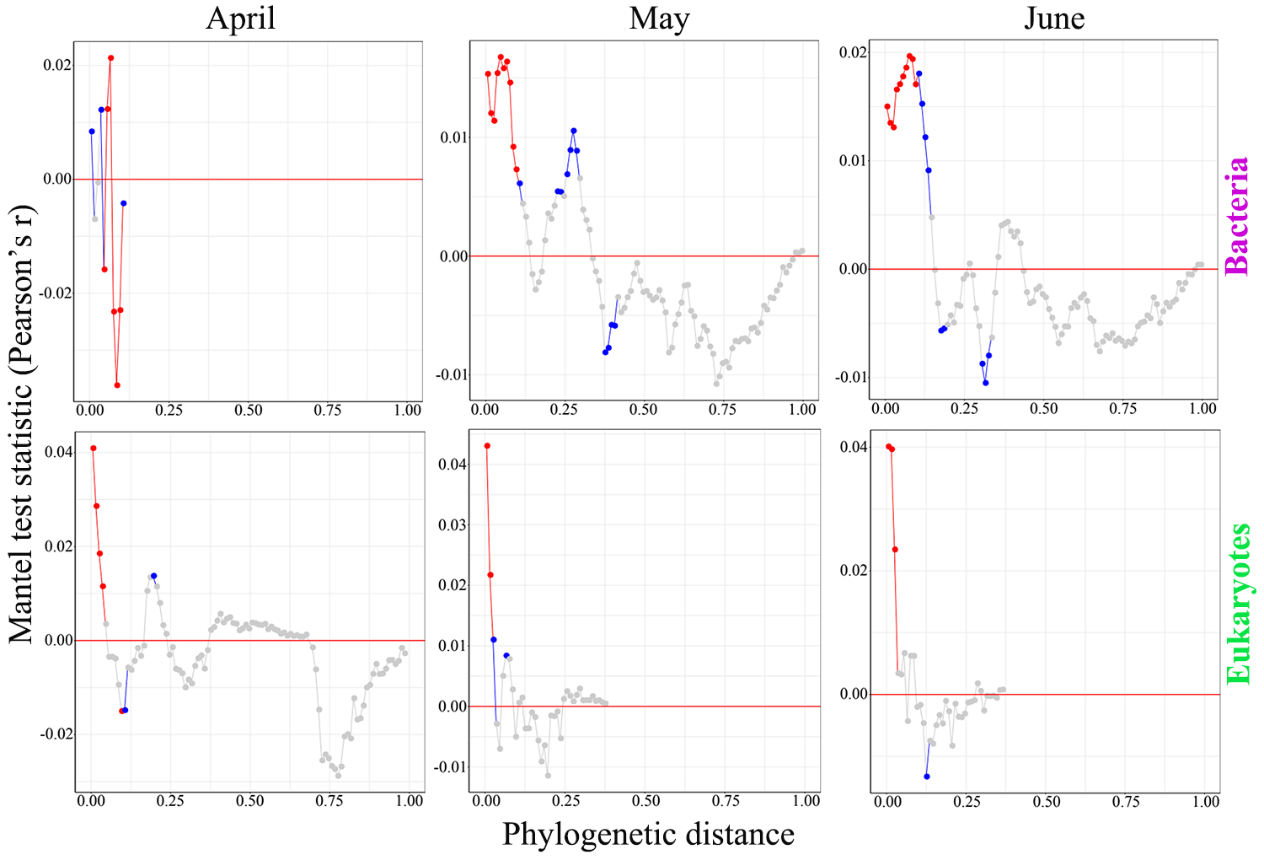


**Figure S10**. Mantel correlogram revealing significant phylogenetic signals of bacterial and eukaryotic communities in lake water occurring at short phylogenetic distances along environmental gradients (i.e., water physicochemical properties) in the three sampling months. Each point represents the Mantel correlation coefficient of the given range in phylogenetic distances. Red, blue, and grey symbols denote highly significant (*p* < 0.01), significant (*p* < 0.05) and insignificant (*p* > 0.05) correlations, respectively


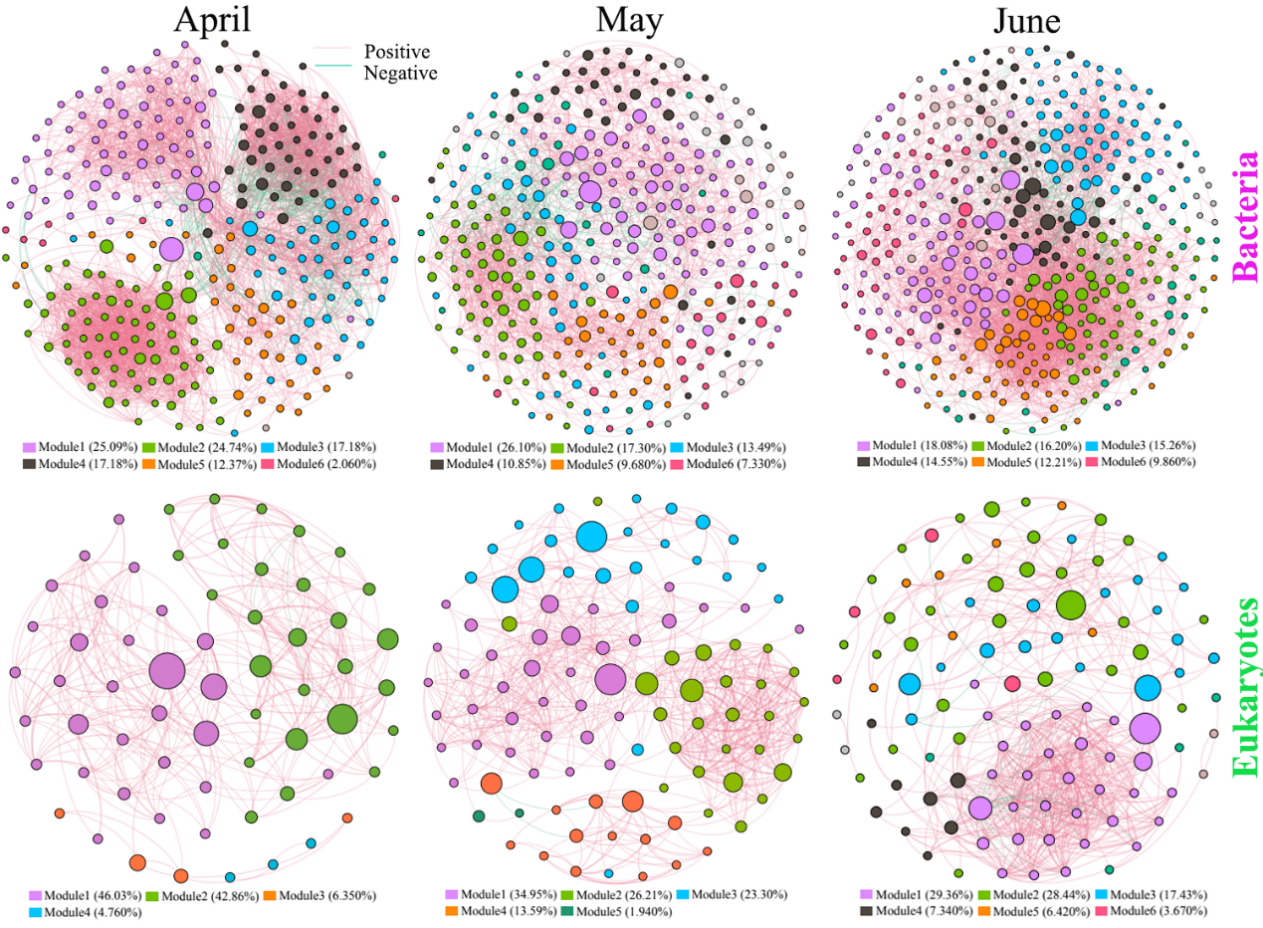


**Figure S11**. Co-occurrence networks of bacteria and eukaryotes in the three sampling months. The nodes (circles) in the networks denote ASVs, and the size of the nodes reflects the value of betweenness centrality. The red and green edges (lines) in the networks represent significant positive and negative correlations, respectively


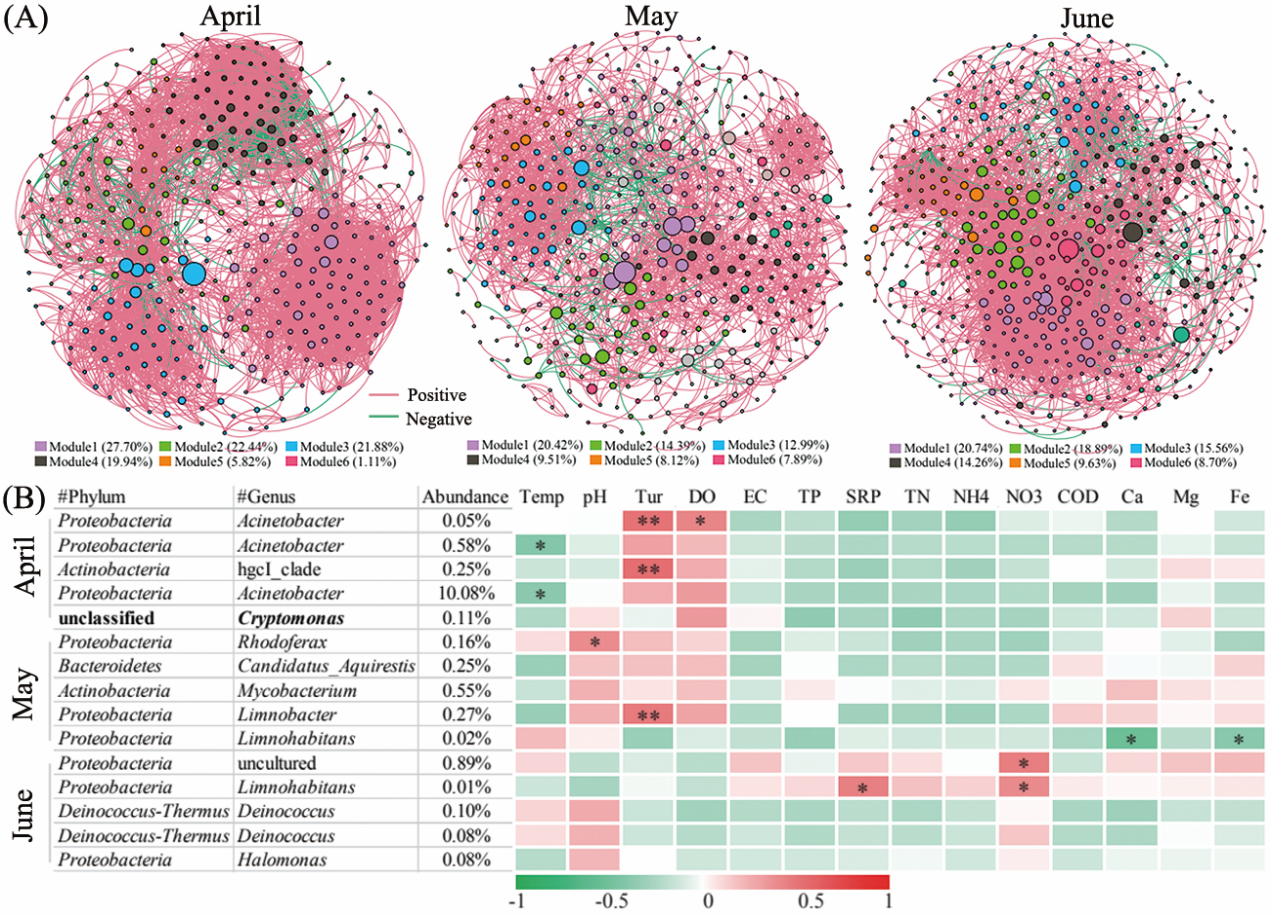


**Figure S12**. Co-occurrence patterns of planktonic community and core species in three months. (A) Co-occurrence networks plankton (bacteria and eukaryote are regarded as a whole) during the sampling period. The nodes (circles) in the networks represent ASVs, and the size of the node reflects the value of betweenness centrality. (B) Relative abundances of the top five core species (those with the highest betweenness centrality in the networks) and their correlations with physicochemical factors. Asterisks denote significance (*, *p* < 0.05; **, *p* < 0.01). Abbreviations: Temp, temperature; DO, dissolved oxygen; Tur, turbidity; EC, electrical conductivity; TP, total phosphorus; SRP, soluble reactive phosphorus; TN, total nitrogen; NH_4_^+^–N, ammonia nitrogen; NO_3_^–^–N, nitrate nitrogen; COD, chemical oxygen demand; Ca, calcium; Mg, magnesium; and Fe, iron.

**Important R codes for statistical analyses**

The R codes are collected from github (<https://github.com/shuojiao/Community-assembly-processes-and-species-coexistences-of-agricultural-soils-microbiome>) and published articles (<https://doi.org/10.1016/j.soilbio.2020.107866>).

**# For null model-based community assembly**

setwd("C:\\Users\\Administrator\\Desktop\\R")

library(picante)

comun=read.csv("OTU.csv",header=T,row.names=1)

comun=t(comun)

phylo=read.tree("Tree.newick")

Beta_NTI<-function(phylo,comun,beta.reps=999){

require(picante)

match.phylo.comun = match.phylo.data(phylo, t(comun))

beta.mntd.weighted = as.matrix(comdistnt(t(match.phylo.comun$data),cophenetic(match.phylo.comun$phy),abundance.weighted=T))

rand.weighted.bMNTD.comp = array(c(-999),dim=c(ncol(match.phylo.comun$data),ncol(match.phylo.comun$data),beta.reps))

for (rep in 1:beta.reps) {

rand.weighted.bMNTD.comp[,,rep] = as.matrix(comdistnt(t(match.phylo.comun$data),taxaShuffle(cophenetic(match.phylo.comun$phy)),abundance.weighted=T,exclude.conspecifics = F))

print(c(date(),rep))

}

weighted.bNTI = matrix(c(NA),nrow=ncol(match.phylo.comun$data),ncol=ncol(match.phylo.comun$data))

for(columns in 1:(ncol(match.phylo.comun$data)-1)) {

for(rows in (columns+1):ncol(match.phylo.comun$data)) {

rand.vals = rand.weighted.bMNTD.comp[rows,columns,];

weighted.bNTI[rows,columns] = (beta.mntd.weighted[rows,columns] - mean(rand.vals)) / sd(rand.vals)

rm("rand.vals")

}

}

rownames(weighted.bNTI) = colnames(match.phylo.comun$data);

colnames(weighted.bNTI) = colnames(match.phylo.comun$data);

return(as.dist(weighted.bNTI))

}

bNTI=Beta_NTI(phylo,comun,beta.reps=999)

write.csv(as.matrix(bNTI),"βNTI.csv")

#RC_bray

raup_crick= function(comun, reps=999){

require(ecodist)

## count number of sites and total species richness across all plots (gamma)

n_sites<-nrow(comun)

gamma<-ncol(comun)

##build a site by site matrix for the results, with the names of the sites in the row and col names:

results<-matrix(data=NA, nrow=n_sites, ncol=n_sites, dimnames=list(row.names(comun), row.names(comun)))

##make the comun matrix into a new, pres/abs. matrix:

ceiling(comun/max(comun))->comun.inc

##create an occurrence vector- used to give more weight to widely distributed species in the null model:

occur<-apply(comun.inc, MARGIN=2, FUN=sum)

##create an abundance vector- used to give more weight to abundant species in the second step of the null model:

abundance<-apply(comun, MARGIN=2, FUN=sum)

##make_null:

##looping over each pairwise community combination:

for(null.one in 1:(nrow(comun)-1)){

for(null.two in (null.one+1):nrow(comun)){

null_bray_curtis<-NULL

for(i in 1:reps){

##two empty null communities of size gamma:

com1<-rep(0,gamma)

com2<-rep(0,gamma)

##add observed number of species to com1, weighting by species occurrence frequencies:

com1[sample(1:gamma, sum(comun.inc[null.one,]), replace=FALSE, prob=occur)]<-1

com1.samp.sp = sample(which(com1>0),(sum(comun[null.one,])-sum(com1)),replace=TRUE,prob=abundance[which(com1>0)]);

com1.samp.sp = cbind(com1.samp.sp,1); # head(com1.samp.sp);

com1.sp.counts = as.data.frame(tapply(com1.samp.sp[,2],com1.samp.sp[,1],FUN=sum)); colnames(com1.sp.counts) = 'counts'; # head(com1.sp.counts);

com1.sp.counts$sp = as.numeric(rownames(com1.sp.counts)); # head(com1.sp.counts);

com1[com1.sp.counts$sp] = com1[com1.sp.counts$sp] + com1.sp.counts$counts; # com1;

#sum(com1) - sum(spXsite[null.one,]); ## this should be zero if everything work properly

rm('com1.samp.sp','com1.sp.counts');

##same for com2:

com2[sample(1:gamma, sum(comun.inc[null.two,]), replace=FALSE, prob=occur)]<-1

com2.samp.sp = sample(which(com2>0),(sum(comun[null.two,])-sum(com2)),replace=TRUE,prob=abundance[which(com2>0)]);

com2.samp.sp = cbind(com2.samp.sp,1); # head(com2.samp.sp);

com2.sp.counts = as.data.frame(tapply(com2.samp.sp[,2],com2.samp.sp[,1],FUN=sum)); colnames(com2.sp.counts) = 'counts'; # head(com2.sp.counts);

com2.sp.counts$sp = as.numeric(rownames(com2.sp.counts)); # head(com2.sp.counts);

com2[com2.sp.counts$sp] = com2[com2.sp.counts$sp] + com2.sp.counts$counts; # com2;

# sum(com2) - sum(spXsite[null.two,]); ## this should be zero if everything work properly

rm('com2.samp.sp','com2.sp.counts');

null.comun = rbind(com1,com2); # null.comun;

##calculate null bray curtis

null_bray_curtis[i] = distance(null.comun,method='bray-curtis');

}; # end reps loop

## empirically observed bray curtis

obs.bray = distance(comun[c(null.one,null.two),],method='bray-curtis');

##how many null observations is the observed value tied with?

num_exact_matching_in_null = sum(null_bray_curtis==obs.bray);

##how many null values are smaller than the observed *dissimilarity*?

num_less_than_in_null = sum(null_bray_curtis<obs.bray);

rc = ((num_less_than_in_null +(num_exact_matching_in_null)/2)/reps)

##modification of raup crick standardizes the metric to range from -1 to 1 instead of 0 to 1

rc = (rc-.5)*2

results[null.two,null.one] = round(rc,digits=2); ##store the metric in the results matrix

print(c(null.one,null.two,date()));

}; ## end null.two loop

}; ## end null.one loop

results<-as.dist(results)

return(results)

}

RC=raup_crick(comun)

write.csv(as.matrix(RC),"Mac-RC.csv")

**# For niche breadth analysis**

library(vegan)

library(spaa)

dt=read.csv("C:/Users/Administrator/Desktop/T5-OTU.csv",header = T,row.names = 1)

dt=t(dt)

niche.width=niche.width(dt,method="levins")

dd=t(niche.width)

write.csv(as.matrix(dd),"T5-niche.csv")

**# For neutral model**

comun=read.csv("C:/Users/Administrator/Desktop/Bare-Fun.csv",header=T,row.names=1)

comun=t(comun)

Neutral.fit <- function(comun, stats=TRUE){

require(minpack.lm)

require(Hmisc)

require(stats4)

options(warn=-1)

#Calculate the number of individuals per community

N <- round(mean(apply(comun, 1, sum)))

#Calculate the average relative abundance of each taxa across communities

p.m <- apply(comun, 2, mean)

p.m <- p.m[p.m != 0]

p <- p.m/N

#Calculate the occurrence frequency of each taxa across communities

comun.bi <- 1*(comun>0)

freq <- apply(comun.bi, 2, mean)

freq <- freq[freq != 0]

#Combine

C <- merge(p, freq, by=0)

C <- C[order(C[,2]),]

C <- as.data.frame(C)

C.0 <- C[!(apply(C, 1, function(y) any(y == 0))),]

p <- C.0[,2]

freq <- C.0[,3]

names(p) <- C.0[,1]

names(freq) <- C.0[,1]

#Calculate the limit of detection

d = 1/N

##Fit model parameter m (or Nm) using Non-linear least squares (NLS)

m.fit <- nlsLM(freq ~ pbeta(d, N*m*p, N*m*(1-p), lower.tail=FALSE), start=list(m=0.1))

m.ci <- confint(m.fit, 'm', level=0.95)

##Fit neutral model parameter m (or Nm) using Maximum likelihood estimation (MLE)

sncm.LL <- function(m, sigma){

R = freq - pbeta(d, N*m*p, N*m*(1-p), lower.tail=FALSE)

R = dnorm(R, 0, sigma)

-sum(log(R))

}

m.mle <- mle(sncm.LL, start=list(m=0.1, sigma=0.1), nobs=length(p))

##Calculate Akaike's Information Criterion (AIC)

aic.fit <- AIC(m.mle, k=2)

bic.fit <- BIC(m.mle)

##Calculate goodness-of-fit (R-squared and Root Mean Squared Error)

freq.pred <- pbeta(d, N*coef(m.fit)*p, N*coef(m.fit)*(1-p), lower.tail=FALSE)

Rsqr <- 1 - (sum((freq - freq.pred)^2))/(sum((freq - mean(freq))^2))

RMSE <- sqrt(sum((freq-freq.pred)^2)/(length(freq)-1))

pred.ci <- binconf(freq.pred*nrow(comun), nrow(comun), alpha=0.05, method="wilson", return.df=TRUE)

##Calculate AIC for binomial model

bino.LL <- function(mu, sigma){

R = freq - pbinom(d, N, p, lower.tail=FALSE)

R = dnorm(R, mu, sigma)

-sum(log(R))

}

bino.mle <- mle(bino.LL, start=list(mu=0, sigma=0.1), nobs=length(p))

aic.bino <- AIC(bino.mle, k=2)

bic.bino <- BIC(bino.mle)

##Goodness of fit for binomial model

bino.pred <- pbinom(d, N, p, lower.tail=FALSE)

Rsqr.bino <- 1 - (sum((freq - bino.pred)^2))/(sum((freq - mean(freq))^2))

RMSE.bino <- sqrt(sum((freq - bino.pred)^2)/(length(freq) - 1))

bino.pred.ci <- binconf(bino.pred*nrow(comun), nrow(comun), alpha=0.05, method="wilson", return.df=TRUE)

##Results

if(stats==TRUE){

fitstats <- data.frame(m=numeric(),m.ci=numeric(), m.mle=numeric(), maxLL=numeric(), binoLL=numeric(), Rsqr=numeric(), Rsqr.bino=numeric(), RMSE=numeric(), RMSE.bino=numeric(), AIC=numeric(), BIC=numeric(), AIC.bino=numeric(), BIC.bino=numeric(), N=numeric(), Samples=numeric(), Richness=numeric(), Detect=numeric())

fitstats[1,] <- c(coef(m.fit), coef(m.fit)-m.ci[1], m.mle@coef['m'], m.mle@details$value, bino.mle@details$value, Rsqr, Rsqr.bino, RMSE, RMSE.bino, aic.fit, bic.fit, aic.bino, bic.bino, N, nrow(comun), length(p), d)

return(fitstats)

} else {

A <- cbind(p, freq, freq.pred, pred.ci[,2:3], bino.pred, bino.pred.ci[,2:3])

A <- as.data.frame(A)

colnames(A) <- c('p', 'freq', 'freq.pred', 'pred.lwr', 'pred.upr', 'bino.pred', 'bino.lwr', 'bino.upr')

B <- A[order(A[,1]),]

return(B)

}

}

Freq=Neutral.fit(comun)

write.csv(as.matrix(Freq),"value.csv")

**#For topological parameters of subnetwork**

library(tibble)

library(ggplot2)

library(ggcor)

library(vegan)

library(dplyr)

library(tidygraph)

library(ggraph)

library(igraph)

library(colormap)

library(wesanderson)

library(reshape2)

library(qgraph)

library(randomcoloR)

sp<-read.csv("OTU.csv",header=TRUE,row.names=1)

sp1=t(sp)

#sp1<-select_if(sp,function(x)!all(x==0))

net <- fast_correlate(sp1,method="spearman",p.adjust=TRUE,p.adjust.method = "fdr") %>%

as_tbl_graph(r.thres = 0.6, p.thres = 0.01) %>%

mutate(degree = tidygraph::centrality_degree(mode = "all")) %>%

activate("edges") %>%

mutate(col = ifelse(r>= 0,alpha("red", 0.1),alpha("blue", 0.1)))%>% as_cor_network()

nodes<-net$nodes

edges<-net$edges

net<- graph_from_data_frame(edges, directed=FALSE, vertices=nodes)

net <- simplify(net)

net <- delete.vertices(net, names(degree(net)[degree(net) == 0]))

Nodes <- Links <- Degrees <- clustering_coefficient <- Closenesses <- Betweennesses <- C <- B<- D<- c(rep(1,15))

nettestt <- data.frame(t(sp1)) %>%

rownames_to_column(var = "SampleID")

for (i in 1:15){

current_net <- subgraph(net, which(V(net)$name %in% as.character(nettestt$SampleID[nettestt[,1+i] != 0])))

Nodes [i] <- mean(length(V(current_net)))

Links [i] <- mean(length(E(current_net)))

Degrees [i] <- mean(igraph::degree(current_net,mode="all"))

clustering_coefficient[i] <- mean(transitivity(current_net))

Closenesses[i] <- mean(closeness(current_net, normalized = TRUE))

Betweennesses[i] <- mean(betweenness(current_net, directed = FALSE, normalized = TRUE))

C[i] <- mean(closeness(current_net))

B[i] <- mean(betweenness(current_net, directed = FALSE))

D[i] <- mean(graph.density(current_net,loop=FALSE))

}

test <- data.frame(cbind(Nodes,Links,Degrees, clustering_coefficient,Closenesses,Betweennesses,C,B,D))

write.csv(test,"index.csv")

**# Phylogenetic signal along environmental analysis**

library(dplyr)

library(picante)

library(ggplot2)

library(vegan)

otu=read.csv("OTU.csv", row.names = 1, header = T)

otu=t(otu)

#apply(otu, 1, sum)

env=read.csv("Physicochemical factor.csv", header = T, row.names = 1)

env=scale(env,center = TRUE, scale = TRUE)

phy=read.tree("Lytree.newick")

# how many tips does our phylogeny have?

Ntip(phy)

# plot(phy)

# check for mismatches/missing species

combined=match.phylo.comm(phy, otu)

# the resulting object is a list with $phy and $comm elements, replace our original data with the sorted/matched data

phy=combined$phy

otu=combined$comm

idx = intersect(rownames(env),rownames(otu))

otu=otu[idx, ] %>% t() %>% as.data.frame()

env=env[idx, ]

result=(as.matrix(otu[1,]) %*% as.matrix(env)) / (sum(otu[1,]))

for(i in 2:nrow(otu)){

tmp <- (as.matrix(otu[i,]) %*% as.matrix(env)) / (sum(otu[i,]))

result <- rbind(result,tmp)

}

#write.csv(result, "Abundant_niche.csv")

niche.eud=vegdist(result, method = "euclidean", upper = FALSE)

phy.dist=cophenetic(phy)

#normalization,scale to 0~1

phy.dist=(phy.dist - min(phy.dist))/ (max(phy.dist) - min(phy.dist))

otu.correlog=mantel.correlog(niche.eud, phy.dist, nperm = 1000, mult = "bonferroni", n.class=100, cutoff = FALSE)

sink('correlog.txt')

dt <- as.data.frame(otu.correlog[["mantel.res"]])

dt1 <- dt[complete.cases(dt),]

fun<-function(x){ifelse(x<0.01,"hs",ifelse(x<0.05,"s",ifelse(x>0.05,"ins","")))}

dt1$pointtyp <- fun(dt1$`Pr(corrected)`)

dt1$linetyp <- "ll"

RS <- ggplot(data=dt1, aes(x=class.index, y=Mantel.cor,color=pointtyp))+

geom_line(aes(group=linetyp))+geom_point(size=5)+

labs(x = "Phylogenetic distances", y = "Mantel test statistic(Pearson's r)")+

theme_bw()+

scale_shape_manual(values = c(1,1,1))+

scale_color_manual(values = c("red ","gray80","blue"))+

theme(axis.title = element_text(size = 18,colour = "black"))+

theme(axis.text = element_text(size = 18,colour = "black"))+

theme(axis.line = element_line(colour = "black",size = 0.6))+

theme(axis.ticks = element_line(colour = "black",size = 0.6))+

theme(axis.text.x = element_text(colour = "black",size = 18))+

theme(axis.text.y = element_text(colour = "black",size = 18))+

theme(strip.text = element_text(face= "bold", size = 16,color="black"))+

geom_hline (yintercept = 0,color="red", size=1)+

xlim(0,1)+theme(legend.position = "none")

ggsave("Figure.pdf",height=12,width=12)

**# For species replacement and richness difference**

library(adespatial)

library(data.table)

library(tidyr)

library(stringr)

library(ggtern)

source("mymultdf.R",encoding = "utf-8")

grp <- read.csv("grp.csv",header = TRUE,row.names = 1)

sps <- read.csv("S16-SHQ.csv",header = T,row.names = 1)

sps=t(sps)

##

btres <- beta.div.comp(sps, coef = 'S', quant = T,save.abc = TRUE)

btres$part

Repl <- as.data.frame(as.matrix(btres$repl))

Repl$st <- row.names(Repl)

Repl <- reshape2::melt(Repl,id.vars="st")

Repl <- Repl["value"]

names(Repl) <- "Repl"

RichDiff <- as.data.frame(as.matrix(btres$rich))

RichDiff$st <- row.names(RichDiff)

RichDiff <- reshape2::melt(RichDiff,id.vars="st")

RichDiff <- RichDiff["value"]

names(RichDiff) <- "RichDiff"

Similarity <- 1-as.data.frame(as.matrix(btres$D))

Similarity$st <- row.names(Similarity)

Similarity <- reshape2::melt(Similarity,id.vars="st")

Similarity <- Similarity["value"]

names(Similarity) <- "Similarity"

btres <- cbind(Repl,RichDiff,Similarity)

dfgrp <- as.data.frame(rep(grp$group,each=nrow(sps)))

names(dfgrp) <- "group"

dfres <- mydffc(mydfdt = btres,grp = dfgrp,mymethod = "LSD",padj = "fdr")

dfres

dfres$dtgrp <- factor(dfres$dtgrp,levels = c("A","B"))

ggplot(dfres,aes(dtgrp,dtmean,fill=dtgrp)) +

geom_bar(stat="identity",show.legend = FALSE)+

geom_text(aes(x = dtgrp,y = (dtmean+ste)*1.05,label = dflab),

size = 5,color = "black")+

geom_errorbar(aes(ymin = (dtmean-ste), ymax = (dtmean+ste)),width = 0.4)+

facet_wrap(.~variable,ncol =4,scales = "free_y")+

labs(x=NULL,y=NULL)+

facet_wrap(.~variable,ncol =4,scales = "free_y")+

# scale_fill_manual(values = c("CK"="#7570B3","NP"="#1B9E77","MNP"="#D95F02","2MNP"="#E7298A"))+

scale_y_continuous(labels = scales::label_comma(accuracy =0.01))+

theme(axis.title = element_text(size = 15,colour = "black"))+

theme(axis.text = element_text(colour = "black",size = 15,angle = 0,vjust = 0.5))+

theme(strip.text = element_text(size = 15),

text = element_text(family = "serif"))

write.csv(as.matrix(btres),"btres.csv")

library(vcd)

grp=read.csv("grp.csv",header = TRUE,row.names = 1)

btres=read.csv("All-btres.csv",header = T,row.names = 1)

btres$grp <- factor(btres$grp,levels = c("A","B","C"))

btres2 <- btres[,c("RichDiff","Similarity","Repl")]

cols <- btres$grp

ternaryplot(btres2,prop_size = 0.1,col = "black",

main=NULL)
